# Novelty detection in an auditory oddball task on freely moving rats

**DOI:** 10.1038/s42003-023-05403-y

**Published:** 2023-10-19

**Authors:** Laura Quintela-Vega, Camilo J. Morado-Díaz, Gonzalo Terreros, Jazmín S. Sánchez, David Pérez-González, Manuel S. Malmierca

**Affiliations:** 1 Cognitive and Auditory Neuroscience Laboratory, Institute of Neuroscience of Castilla y León, Calle Pintor Fernando Gallego 1, 37007 Salamanca, Spain; 2 The Salamanca Institute for Biomedical Research (IBSAL), 37007 Salamanca, Spain; 3 Instituto de Ciencias de la Salud. Universidad de O´Higgins, Rancagua, Chile; 4 Department of Biology and Pathology, Faculty of Medicine, Campus Miguel de Unamuno, University of Salamanca, 37007 Salamanca, Spain; 5 Department of Basic Psychology, Psychobiology and Methodology of Behavioural Sciences. Faculty of Psychology, University of Salamanca, 37005 Salamanca, Spain

**Keywords:** Auditory system, Cognitive neuroscience

## Abstract

The relative importance or saliency of sensory inputs depend on the animal’s environmental context and the behavioural responses to these same inputs can vary over time. Here we show how freely moving rats, trained to discriminate between deviant tones embedded in a regular pattern of repeating stimuli and different variations of the classic oddball paradigm, can detect deviant tones, and this discriminability resembles the properties that are typical of neuronal adaptation described in previous studies. Moreover, the auditory brainstem response (ABR) latency decreases after training, a finding consistent with the notion that animals develop a type of plasticity to auditory stimuli. Our study suggests the existence of a form of long-term memory that may modulate the level of neuronal adaptation according to its behavioural relevance, and sets the ground for future experiments that will help to disentangle the functional mechanisms that govern behavioural habituation and its relation to neuronal adaptation.

## Abstract (web summary)

Rats can detect frequency deviant tones in a context-dependent manner, indicating a memory-based adaptation in auditory neurons that is shaped by stimuli saliency and behavioral relevance.

## Introduction

Animals are constantly receiving acoustic information from the environment, most of which can be, and needs to be, ignored. But the detection and selection of novel or salient information can be a matter of life or death. The salience of sensory input depends on the animal’s environmental context and, as a result, the behavioural actions in response to the same inputs can vary with time and experience. One of the most basic forms of learning across organisms and sensory systems is behavioural habituation^1–3^; i.e., a decrease in response to a particular input or environment over repeated exposure, an effect that does not involve neuronal fatigue^3–6^. However, habituated behavioural responses can be reversed by the addition of new sensory stimuli. The detection of these novel stimuli can be innate or shaped by experience. For example, the cortical representation of pup calls in mothers is enhanced when compared to virgin females and provides for better detection of these sudden and highly behavioural relevant sounds^7,8^. In this scenario, deviance detection, the detection of physical or temporal salient stimuli, may prove more useful^5,9^.

Previous studies carried out using single unit recordings in different animal models have demonstrated that individual neurons along the auditory pathway show stimulus-specific adaptation (SSA)^10–24^. Interestingly, neuronal SSA resembles behavioural habituation in many respects^5^, such as how neurons show a reduction of their response to repeated sounds (standards) that is resumed when a new sound (deviant) occurs. SSA has been found in the auditory midbrain and up to the cortex, and across species and arousal states^5,10,11,17–20,23,25–30^. SSA has been shown to occur on the single-unit level, and thus, can be thought of as a simple, intrinsic property of single neurons. However, given the similarities between neuronal SSA and behavioural habituation, it could be possible that neuronal SSA interacts with, and may be part of, the brain’s memory systems. As such, SSA, while reflecting a kind of learning, may also itself be able to be modulated by learned information. Except for a few previous reports^31–36^, virtually all studies on SSA have been performed using sounds that did not carry any behavioural meaning. Interestingly, neurons in the thalamic reticular nucleus^37,38^, amygdala, and prefrontal cortex^39^, which are involved in selective attention^40^, also show SSA. This suggests that associative learning may be able to modulate the levels of neuronal SSA. For example, using a tone of a particular frequency as a conditioned stimulus (*CS*) in an associative learning paradigm will lead to a shift of the neuronal frequency tuning towards that tone frequency^33,41,42^.

In this work, we implement a set of behavioural tests to investigate the correlation between behavioural habituation and SSA, as described in animal models^10–12,24,43^. We trained freely moving rats to discriminate deviant tones embedded in an oddball paradigm. Our results demonstrate that trained animals were able to recognize violations of the regularity established by repetitive standard tones, in different variations of an oddball paradigm, similar to what occurs at neuronal SSA. Our study implies the existence of a form of long-term memory trace that may modulate the level of neuronal adaptation according to its behavioural relevance, and set the ground for future experiments that will test the potential relationship between neuronal SSA and behavioural habituation.

## Results

**Fig. 1:**
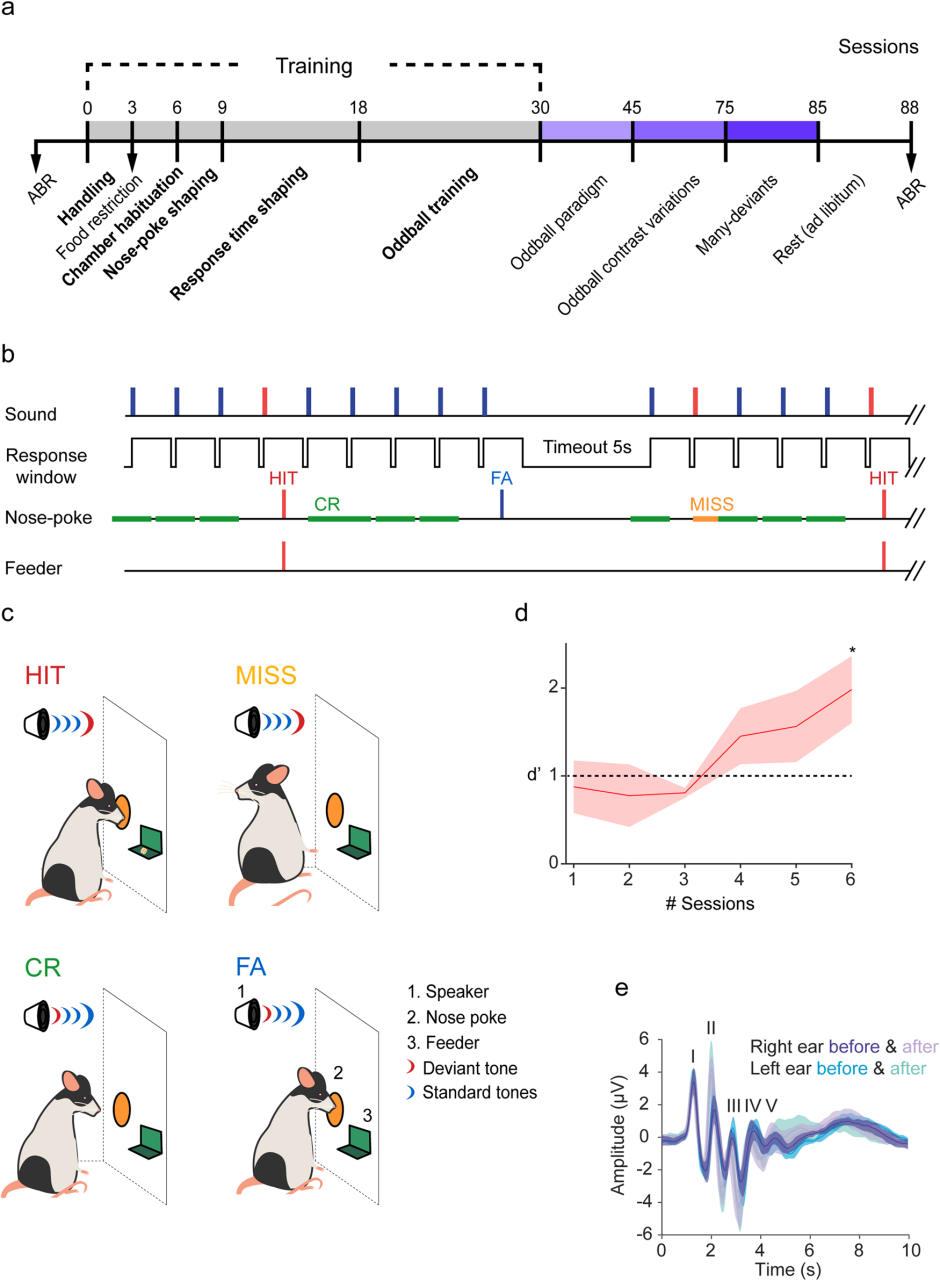
Experimental design. **a** Schematic representation of training/test sessions scheduled across the course of the experiment. Training was divided in 5 different stages, highlighted in bold. **b** Temporal structure of behavioural trials. **c** Cartoons showing all possible responses of the animals inside the operational chamber during behavioural sessions equipped with a speaker (1) to display sound sequences (red wave, deviant sound, blue wave, standard sound). Nose-poke (2) responses in the response window after a deviant tone (red wave) were rewarded with one pellet (3) and considered as Hits (HIT). The absence of response to deviant tone was computed as a Missed response (MISS). Correct rejections were absence of response after a standard tone (CR), and False alarms (FA) were responses to a standard tone (blue wave; followed by 5 s of timeout). **d** Learning curve representing mean *d’* values (red line) ± S.E.M. (pink shaded area) computed for trained rats in the last 6 sessions of the training oddball phase. Rats completed training when they reached criterion performance (*d’* ≥ 1) for 3 consecutive sessions (asterisk). **e** Mean ± S.D.M. auditory brainstem responses (ABR) recorded prior to initiating and after completion of behavioural sessions for both ears (right, in purple; left, in blue). Significant differences were found in the response latency: shorter after the training (three-way ANOVA test for repeated measures for the variables Wave amplitude and Response latency, *p* = 0.001).

**Fig. 2:**
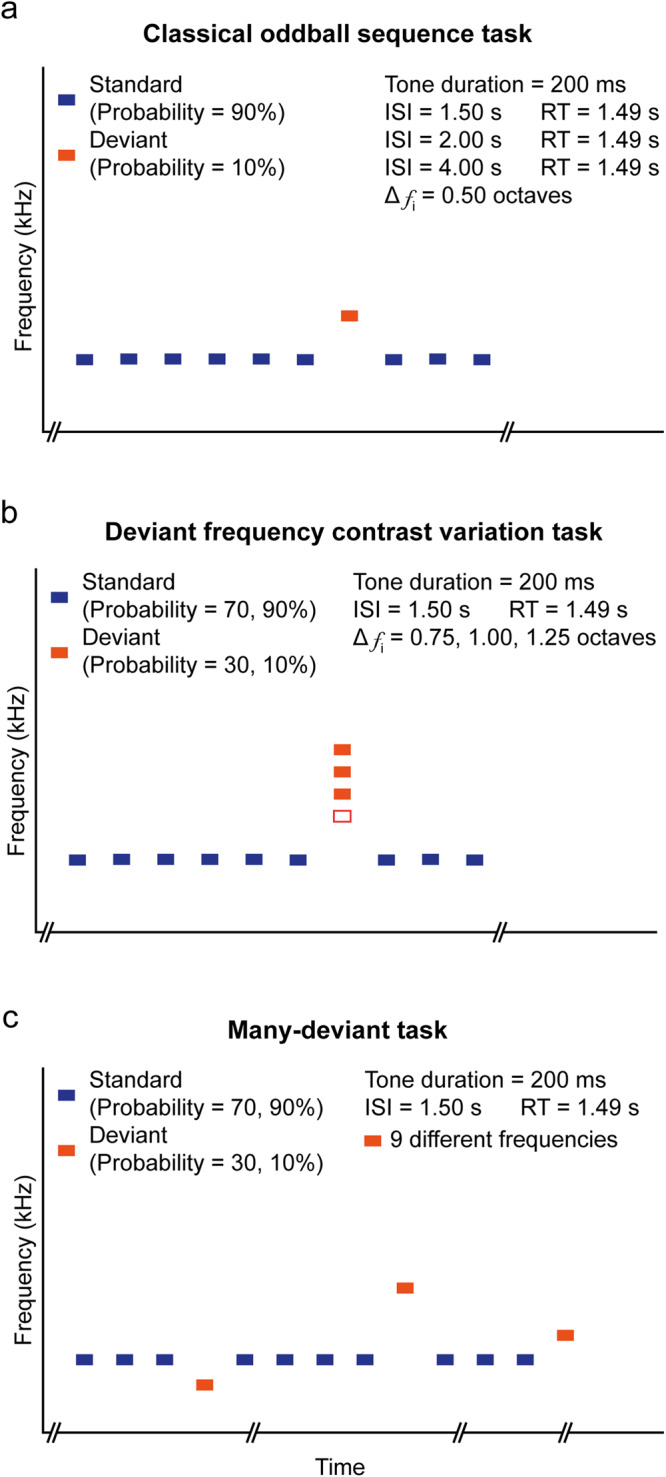
Paradigm sequences. **a** Classical oddball sequence task consisting of a high probability standard tones (90%; in blue) that is randomly interrupted by low probability deviant tones (10%; in red) and 0.5 octaves in frequency contrast (Δ f_i_). Tone duration was 200 ms, the response time (RT) was maintained at 1.49 s and the interstimulus interval (ISI) was varied from 1.5 s, for five sessions, to 2 s for another five sessions and 4 s for the last five sessions. **b** Deviant frequency contrast variation task was similar to the classical oddball paradigm, but here, the frequency contrast between standard/deviant tones varied in 5 different sessions each, being 0.75 octaves for the first five, 1.00 octaves for another five and 1.25 octaves for the final five sessions. These three modifications were then tested under a standard/deviant probability of 70/30% for another five sessions for each frequency contrast applied. **c** Many-deviant task consisted of a many-deviant sequence made of several blocks of the oddball paradigm as in (**a**), where the sound frequency of the deviant tone was randomly changing from 9 possibilities of different tones, while the standard sound was maintained throughout the entire sequence. This sequence was also tested under the standard/deviant probability of 70/30%.

We trained eight freely moving rats using different auditory discrimination tasks (Fig. 1a) to examine whether repeated exposure to relevant and/or irrelevant sounds can affect the long-term representation of those sounds in the auditory system. Briefly, we presented a classical oddball paradigm, which consists of a sequence of two different pure tones to the rats, where a low probability sound (deviant, 10% occurrence) was embedded in a high probability pure tone (standard, 90% occurrence). In the first phase of training, the two pure tones were separated by 0.5 octaves. To receive a food reward, animals were required to make a nose-poke response in the operant chamber when a deviant tone (*CS*+) was presented, and to ignore the standard tones (*CS*−, Fig. 1b). Responses were categorized as correct responses (HIT), false alarms (FA), correct rejections (CR) and missed responses (MISS; Fig. 1c) and evaluated with the *d’* sensitivity index, with larger *d’* values indicating better discriminatory ability. Animals were randomly assigned to 2 different groups according to the frequency pair used for the behavioural tasks: 4.8–6.7 kHz and 8.0–11.3 kHz (4 rats each). One of the animals died due to an adverse reaction during anaesthesia for an ABR (auditory brainstem response) test and it only performed the paradigms with a standard/deviant probability of 70/30%. For each group, we used different pairs of sound frequencies, but the standard tone was kept constant per group throughout all the sessions and across the different tasks (Supplementary Table 1). Once rats showed a performance score of *d’* ≥ 1 for 3 consecutive sessions (Fig. 1d), animals were ready for data collection using a set of the different variants of the auditory discrimination task (Fig. 2). More specifically, we tested (1) frequency contrast, (2) the probability of occurrence and (3) the effect of interstimulus interval (stimulus presentation rate). The training process was tailored for each animal according to its learning rate but, on average, the whole process lasted ~48 ± 4 sessions. To show that rats had normal hearing through all the behavioural experiments, ABR-tests^44^ were conducted in all animals both before the training started and after the end of the experiment (Fig. 1e). The analysis (three-way ANOVA test for repeated measures with Group, Time and Ear as factors, Holm-Sidak method for multiple comparisons; Supplementary Table 2) of the I-V waves amplitude showed no significant differences between experimental groups (F_(1,5)_ = 0.869, *p* = 0.384) or before and after the training (F_(1,5)_ = 2.503, *p* = 0.174). Interestingly, the latency of waves I-V of the response after the training were significantly lower (F_(1,5)_ = 60.044, *p* = 0.001), although there were no differences between the experimental groups (F_(1,5)_ = 0.030, *p* = 0.870). This difference in the response latency, after the training period concluded, suggests that there might be some plasticity affecting the response to auditory stimuli in general.

In the following, first, we will demonstrate behavioural evidence that freely-moving rats can successfully detect deviant sounds under the classical oddball paradigm with different variations in the interstimulus interval. Next, we show how this performance is influenced by the frequency contrast between the deviant and standard tones, tested under two different standard/deviant probabilities. Finally, we will show that animals can detect violations in a regular sequence of standard tones using different deviant tones irrespective of their frequency, tested under two different standard/deviant probabilities.

### Behavioural responses to the oddball paradigm

**Fig. 3:**
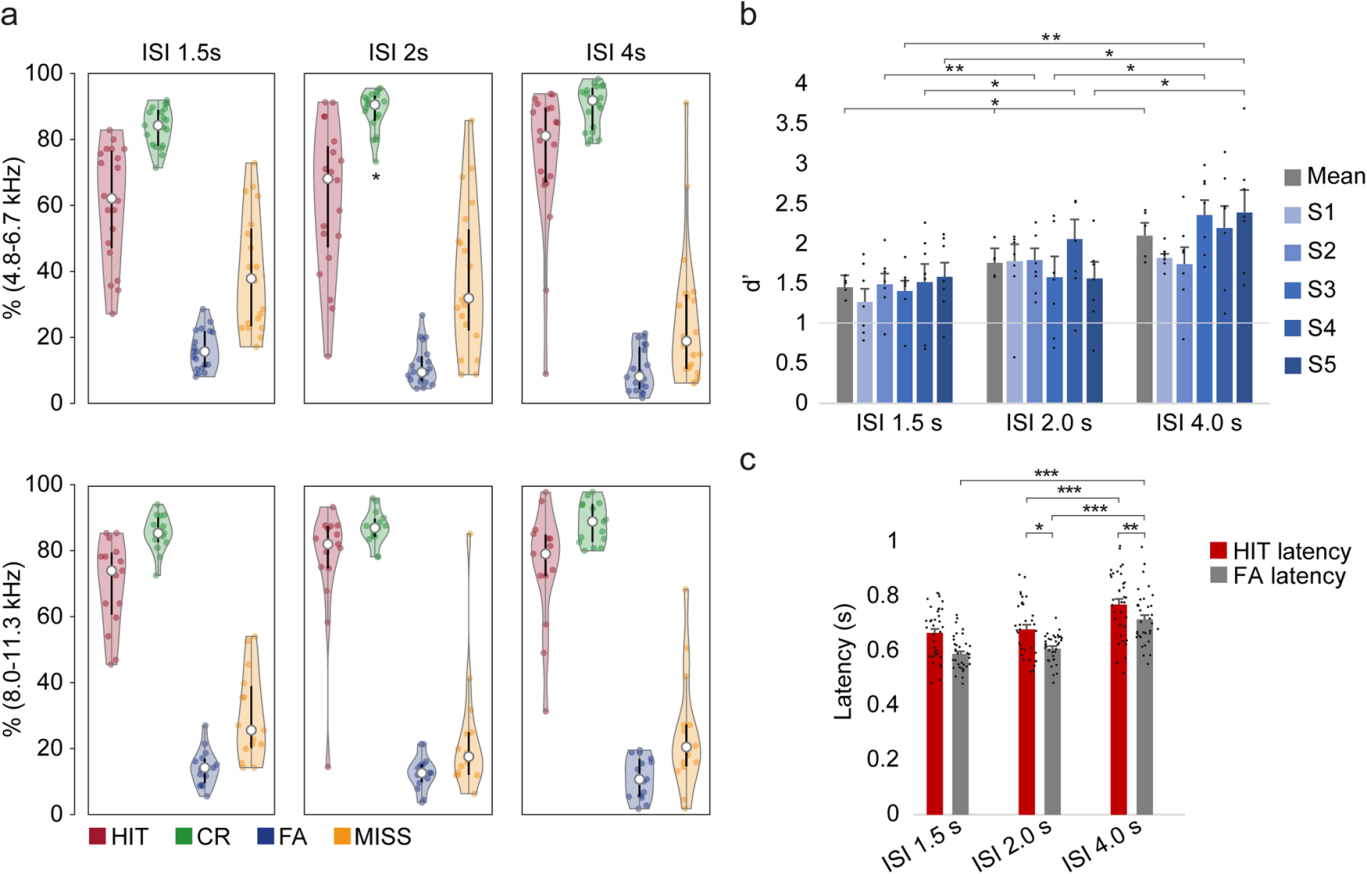
Behavioural responses to the oddball sequence task. **a** Violin plots showing the distribution of averaged percentage (across 5 sessions/animal) of hits after a deviant tone (HIT, in red), correct rejection of standard tones (CR, in green), false alarms (FA, in blue) and missed responses after a deviant tone (MISS, in yellow) performed by trained animals distributed in two groups (4 rats/group) made by the pair of standard-deviant tone frequencies used during training and presented in these oddball paradigms (4.8–6.7 kHz, 8.0–11.3 kHz) for each of the interstimulus interval (ISI) presented (1.5, 2, 4 s). The thick black bars expand from the first (Q1) to the third quartile (Q3), and the whiskers show the range of lower and higher adjacent values. Asterisks (*) represent significant differences with respect to the ISI of 1.5 s (*p < 0.05, **p < 0.01, ***p < 0.001; three-way ANOVA test for repeated measures, *p* < 0.05). **b** Bar chart showing *d’* values (mean ± S.E.M. of all animals per group) obtained for sessions 1, 2, 3, 4, 5 (S1–S5) and the averages of them (Mean). Note that all of the animals present a *d’* > 1, the set discrimination threshold (grey line). Asterisks (*) represent significant differences with respect to the ISI of 1.5 s and the ISI of 2 s (*p < 0.05, **p < 0.01, ***p < 0.001; three-way ANOVA test for repeated measures, *p* < 0.05). **c** Bar chart showing the mean latencies for the HIT (in red) and the FA (in grey) responses, for each ISI presented. Asterisks (*) represent significant differences with respect to the ISI of 1.5 s, the ISI of 2 s and between FA and HIT, respectively (*p < 0.05, **p < 0.01, ***p < 0.001; three-way ANOVA test for repeated measures, *p* < 0.05).

To test if trained rats performed better at discrimination of deviant tones as a function of the interstimulus interval (ISI), we presented the oddball paradigm (Fig. 2a) varying the ISI, and testing 3 different durations (1.5, 2 and 4 s). Data was acquired for 15 sessions per animal (5 for each length of the ISI), in which animals were presented with the corresponding pair of tone frequencies (these frequencies varied with the group they belong to, see Supplementary Table 1). All trained rats showed a similar pattern of response (Fig. 3a) with a high percentage of hits and correct rejections, where the larger the ISI the larger the HIT and CR percentage. Thus, if the ISI was 1.5 s, the mean ± SEM percentage values for HITs and CRs for all rats in all trained groups were 64.7 ± 2.8% and 84.6 ± 1.0%, respectively (60.6 ± 3.9%/83.8 ± 1.3% and 70.2 ± 3.5%/85.7 ± 1.4% for groups 4.8–6.7 kHz and 8.0–11.3 kHz). The percentages of HITs and CRs for all animals for an ISI of 2 s were 68.1 ± 3.7%/88.1 ± 1.0% (61.7 ± 5.0%/88.6 ± 1.4% and 76.6 ± 5.0%/87.4 ± 1.3% for groups 4.8–6.7 kHz and 8.0–11.3 kHz), and for the 4 s ISI they were 74.9 ± 3.3%/89.4 ± 1.1% (74.4 ± 4.8%/89.9 ± 1.5% and 75.5 ± 4.4%/88.8 ± 1.6% for groups 4.8–6.7 kHz and 8.0–11.3 kHz) respectively (three-way ANOVA tests for repeated measures of %HIT and %CR with Session, ISI and Group as factors, %CR F_ISI_ = 8.733, *p* = 0.006; Fig. 3a and Supplementary Table 3).

When the responses of these trained animals were computed as *d’* values, they were all larger than the discrimination threshold (*d´* = 1; Fig. 3b), and progressively increased as a function of the ISI tested, with a significantly higher value for the ISI of 2 s (*p* = 0.046) and 4 s (*p* = 0.028) in respect to the 1.5 s ISI. For all trained rats the average *d’* value for the ISI of 1.5 s was 1.5 ± 0.1 (1.3 ± 0.1 and 1.7 ± 0.1 for groups 4.8–6.7 kHz and 8.0–11.3 kHz), for the ISI of 2 s *d’* = 1.8 ± 0.1 (1.6 ± 0.1 and 2.0 ± 0.1 for groups 4.8–6.7 kHz and 8.0–11.3 kHz). Finally, for the ISI of 4 s it was 2.1 ± 0.1 (2.1 ± 0.1 and 2.1 ± 0.2 for groups 4.8–6.7 kHz and 8.0–11.3 kHz; three-way ANOVA test for repeated measures, with Session, ISI and Group as factors, F_ISI_ (2,10) = 12.057, *p* = 0.002; see Fig. 3b and Supplementary Table 3 for post-hoc Holm-Sidak method for multiple comparisons, *p* < 0.05).

An important issue to consider is whether our results may reflect a genuine SSA correlate or a classical frequency discrimination task. Hence, in order to test whether the rats reached a plateau in their discrimination rate in the oddball test on the first day after completing the training period, or if they were still learning the task and improving their performance, we compared the *d’* values obtained across the 5 consecutive sessions. The comparison of averaged *d’* values across sessions revealed no significant differences between the first and the fifth session (F_Session_ (4,20) = 2.275, *p* = 0.097, see Supplementary Table 3 for statistical results). These results demonstrate that the success in discrimination is context dependent, and animals maintain their performance without additional improvement once they reach a certain level. This suggests a deviant detection rather than a simple frequency discrimination.

To evaluate the reaction time of the animals to detect a deviant tone (HIT) we evaluated the latency of the responses for both the HIT (when the animals respond to a deviant, *CS*+ tone) and the FA (when the animals respond to a standard tone, *CS*- tone) under the different manipulations of the oddball paradigm presented. Note that the response window starts from the beginning of the tone to 1.49 s. The results show a significant increase in the reaction time of the animals for the detection of a deviant tone when comparing the ISI of 2 s with the ISI of 4 s (three-way ANOVA tests for repeated measures of HIT latency with Session, ISI and Group as factors, F_ISI_ (2,10) = 7.937, *p* = 0.009; post-hoc Holm-Sidak method for multiple comparisons, *p* < 0.05; Fig. 3c and Supplementary Table 3). The same occurs with the latency of the FA, with a longer response latency the higher the ISI (three-way ANOVA tests for repeated measures of FA latency with Session, ISI and Group as factors, F_ISI_ (2,10) = 80.281, *p* < 0.001; post-hoc Holm–Sidak method for multiple comparisons, *p* < 0.05; Fig. 3c and Supplementary Table 3).

To assess whether animals have a shorter reaction time when responding to a deviant tone (HIT) compared to when responding to a standard tone (FA), we compared the response latency for both situations in each of the paradigms presented. For a 1.5 s ISI there were no significant differences with a mean response latency for the HIT of 0.68 ± 0.02 s, slightly higher than the latency for the FA (0.60 ± 0.01 s). For the 2 s ISI, there were significant differences (*p* = 0.017), showing a mean response latency of 0.69 ± 0.02 s for the HIT and 0.62 ± 0.01 s for the FA. Finally, when the ISI was 4 s the response latency for the HIT was 0.78 ± 0.02 s, significantly higher (*p* = 0.001) than the response latency for the FA (0.73 ± 0.02 s; three-way ANOVA tests for repeated measures of HIT latency and FA latency with Session, Response and Group as factors; post-hoc Holm-Sidak method for multiple comparisons; Fig. 3c and Supplementary Table 4). These results show that animals exhibit a shorter response latency for FA compared to HITs. This phenomenon can be attributed to the expectation generated by the sequence of standard tones, which elicits a response to a standard tone while awaiting a deviant tone. Conversely, the response latency to a deviant tone is prolonged, as it is distinct from the preceding standard tones.

### Frequency contrast impact on behavioural responses to the oddball paradigm

**Fig. 4:**
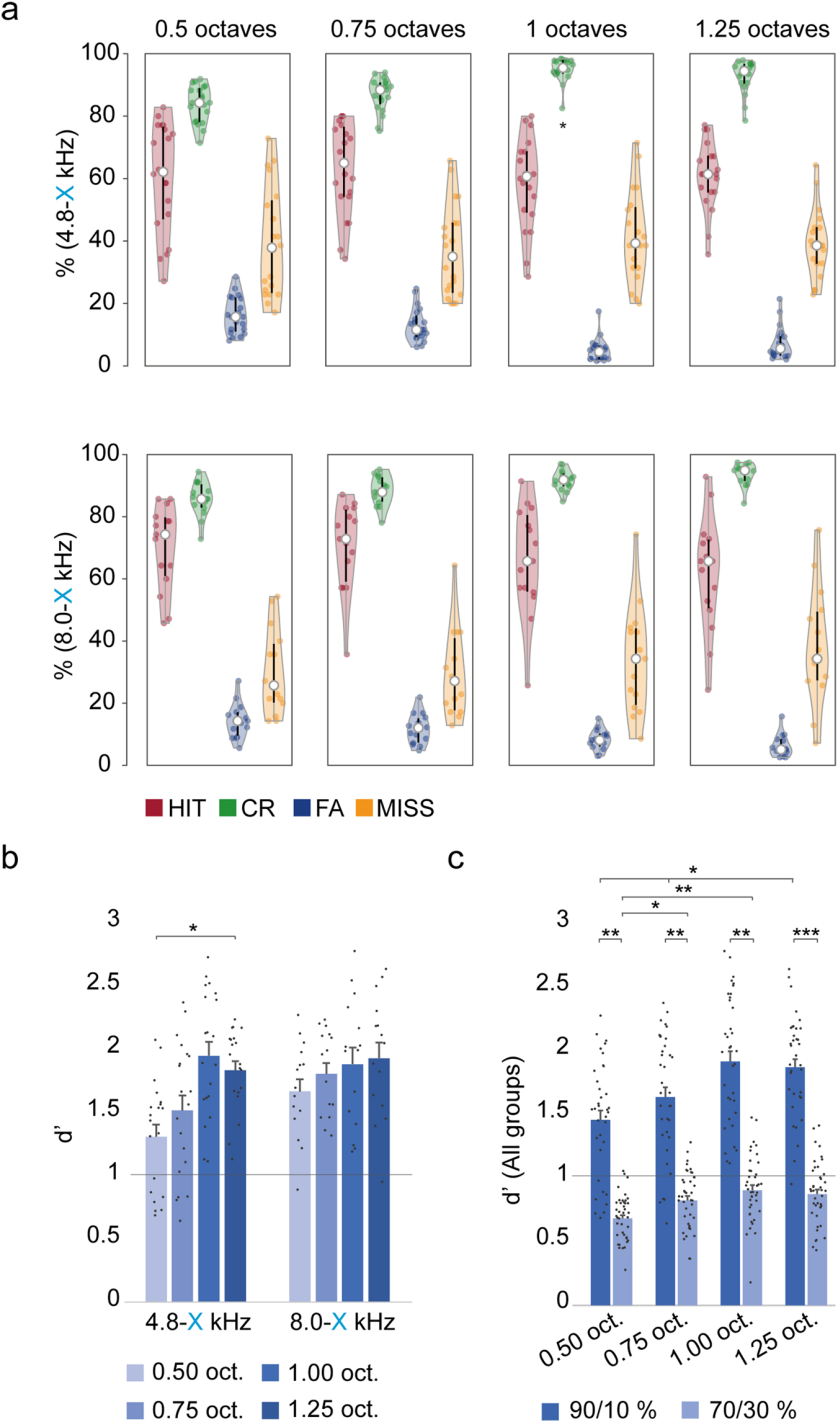
Behavioural responses to oddball sequence task varying frequency contrast. **a** Representation of percentage of hits after a deviant tone (HIT, in red), correct rejection of standard tones (CR, in green), false alarms (FA, in blue) and missed responses after a deviant tone (MISS, in yellow) performed by trained animals distributed in different groups (4 rats/group) made by the pair of standard-deviant tone frequencies (frequency contrast in 0.5 octaves) used during training (4.8–6.7 kHz, 8.0–11.3 kHz). Frequency contrast was varied to 0.75, 1.00 and 1.25 octaves modifying the deviant tone frequency, the ISI was 1.5 s and the standard/deviant probability was 90/10%. Asterisks (*) represent significant differences with respect to the Contrast of 0.50 octaves (*p < 0.05, **p < 0.01, ***p < 0.001; three-way ANOVA test for repeated measures, *p* < 0.05). **b** Bar chart showing *d’* values (mean ± S.E.M. of all animals per group) obtained after frequency contrasts of 0.50, 0.75, 1.00 and 1.25 octaves. Note that for *d’* values of trained animals were *d’* > 1, the set discrimination threshold stablished (grey line). Asterisks (*) represent significant differences with respect to the Contrast of 0.50 octaves (*p < 0.05, **p < 0.01, ***p < 0.001; three-way ANOVA test for repeated measures, *p* < 0.05). **c** Bar chart showing *d’* values (mean ± S.E.M. of all animals) obtained after frequency contrasts of 0.50, 0.75, 1.00 and 1.25 octaves with both Std./Dev. probabilities tested. Note that all of the animals present a *d’* > 1, the set discrimination threshold (dashed line). Asterisks (*) represent significant differences with respect to the Contrast of 0.50 octaves with a probability of 90/10%, the Contrast of 0.50 octaves with a probability of 70/30% and the difference between the two probabilities tested, respectively (*p < 0.05, **p < 0.01, ***p < 0.001; three-way ANOVA test for repeated measures, *p* < 0.05).

To determine whether trained rats performed better at the discrimination of deviant, *CS*+ tones, as a function of the frequency contrast relative to the standard, *CS*- tone, we presented a modification to the oddball paradigm where the standard tone was maintained but the sound frequency of the deviant tone was increased in quarter octave steps from the original deviant frequency (i.e., 0.75, 1.00 and 1.25 octaves) with an ISI of 1.5 s (Fig. 2b). Results were obtained after 5 consecutive sessions for each frequency contrast presented. The performance in all trained groups was similar to the previous task, with high percentage of hits and correct rejections, and low levels of false alarms and missed responses in the 3 frequency contrasts tested (Fig. 4a). Thus, if the frequency contrast between standard and deviant was 0.75 octaves, the mean ± SEM percentage values for HITs and CRs for all rats in all trained groups were 66.4 ± 2.5% and 87.5 ± 0.9%, respectively (63.3 ± 3.3%/86.9 ± 1.2% and 70.6 ± 3.6%/88.3 ± 1.2% for groups 4.8-X kHz and 8.0-X kHz). The percentages of HITs and CRs for a frequency contrast of 1.00 octave were 61.7 ± 2.7% / 93.6 ± 0.7% (58.5 ± 3.2%/95.0 ± 0.8% and 66.0 ± 4.4%/91.8 ± 0.8% for groups 4.8-X kHz and 8.0-X kHz), and for 1.25 octaves they were 61.2 ± 2.4% / 93.2 ± 0.8% (60.9 ± 2.4%/92.8 ± 1.2% and 61.6 ± 4.7%/93.8 ± 0.9% for groups 4.8-X kHz and 8.0-X kHz) respectively. These results show an upward trend in the percentage of correct rejections as the contrast between standard and deviant frequencies increases, while the HITs remain constant (three-way ANOVA tests for repeated measures of %HIT, %CR with Session, Contrast and Group as factors, for the %CR F_Contrast_ (3,15) = 14.535, *p* < 0.001; post-hoc Holm–Sidak method for multiple comparisons, *p* < 0.05; Fig. 4a and Supplementary Table 5). When the responses of these trained animals were computed as *d’* values, they were all larger than 1 (Fig. 4b), and progressively increased as a function of the frequency contrast used being 1.6 ± 0.1 (1.5 ± 0.1 and 1.8 ± 0.9 for groups 4.8-X kHz and 8.0-X kHz) for the 0.75 octaves contrast, 1.9 ± 0.1 (2.0 ± 0.1 and 1.9 ± 0.1 for groups 4.8-X kHz and 8.0-X kHz) for the contrast of 1.00 octaves and 1.9 ± 0.1 (1.8 ± 0.7 and 1.9 ± 0.1 for groups 4.8-X kHz and 8.0-X kHz) for the 1.25 octaves (Fig. 4c). The mean ± SEM *d’* of trained animals using a half octave frequency contrast (oddball paradigm; Fig. 3b) was 1.5 ± 0.1, a significantly lower value than presenting larger frequency contrasts (three-way ANOVA test for repeated measures with Session, Contrast and Group as factors, F_Contrast_ (3,15) = 7.479, *p* = 0.003; post-hoc Holm–Sidak method for multiple comparisons, *p* < 0.05; Supplementary Table 5). Thus, these data suggest that rats discriminate deviant tones more accurately when these tones are more separated in frequency from the standard tone.

To test the ability of rats to discriminate deviant, *CS*+ tones as a function of the probability of occurrence of the standard, *CS*- tone, we presented the 4 different frequency contrasts, changing the standard/deviant probability from 90/10% to 70/30%. Again, the results were obtained after 5 consecutive sessions for each frequency contrast presented. The performance in both trained groups was profoundly affected by this modification, showing an important decline compared to the previous task, with a low percentage of hits, but a high percentage of correct rejections. Thus, if the frequency contrast between standard and deviant tones was 0.50 octaves, the mean ± SEM percentage values for HITs and CRs for all rats in all trained groups were 24.5 ± 1.2% and 91.4 ± 0.6%, respectively. The percentages of HITs and CRs for a frequency contrast of 0.75 octaves were 24.83 ± 1.24% / 93.3 ± 0.5%, for the contrast of 1.00 octave were 29.2 ± 1.4% / 92.2 ± 0.6%, and for the 1.25 octaves these values were 30.3 ± 1.0% / 91.5 ± 0.6%, respectively (three-way ANOVA test for repeated measures of %HIT, %CR with Session, Contrast and Group as factors, F_Contrast_ (3,18) = 6.690, *p* = 0.003; post-hoc Holm–Sidak method for multiple comparisons, *p* < 0.05; Supplementary Table 6). When the responses of these trained animals were computed as *d’* values, they were all significantly lower than with the previous standard/deviant probability tested (90/10%; three-way ANOVA test for repeated measures for each contrast tested with Session, Probability and Group as factors, *p* < 0.05; Supplementary Table 7), and progressively increased as a function of the frequency contrast used (0.7 ± 0.03, 0.8 ± 0.04, 0.9 ± 0.04 and 0.9 ± 0.04 for 0.50, 0.75, 1.00 and 1.25 octaves, respectively Fig. 4c; three-way ANOVA test for repeated measures with Session, Contrast and Group as factors, F_Contrast_ (3,18) = 8.783, *p* = 0.001; post-hoc Holm–Sidak method for multiple comparisons, *p* < 0.05; Supplementary Table 6). Similarly, to what occurred with the 90/10% probability, rats discriminate deviant tones more accurately under the 70/30% probability when the frequency contrast between the standard and deviant tones is larger.

### Responses to the many-deviant sequence

**Fig. 5:**
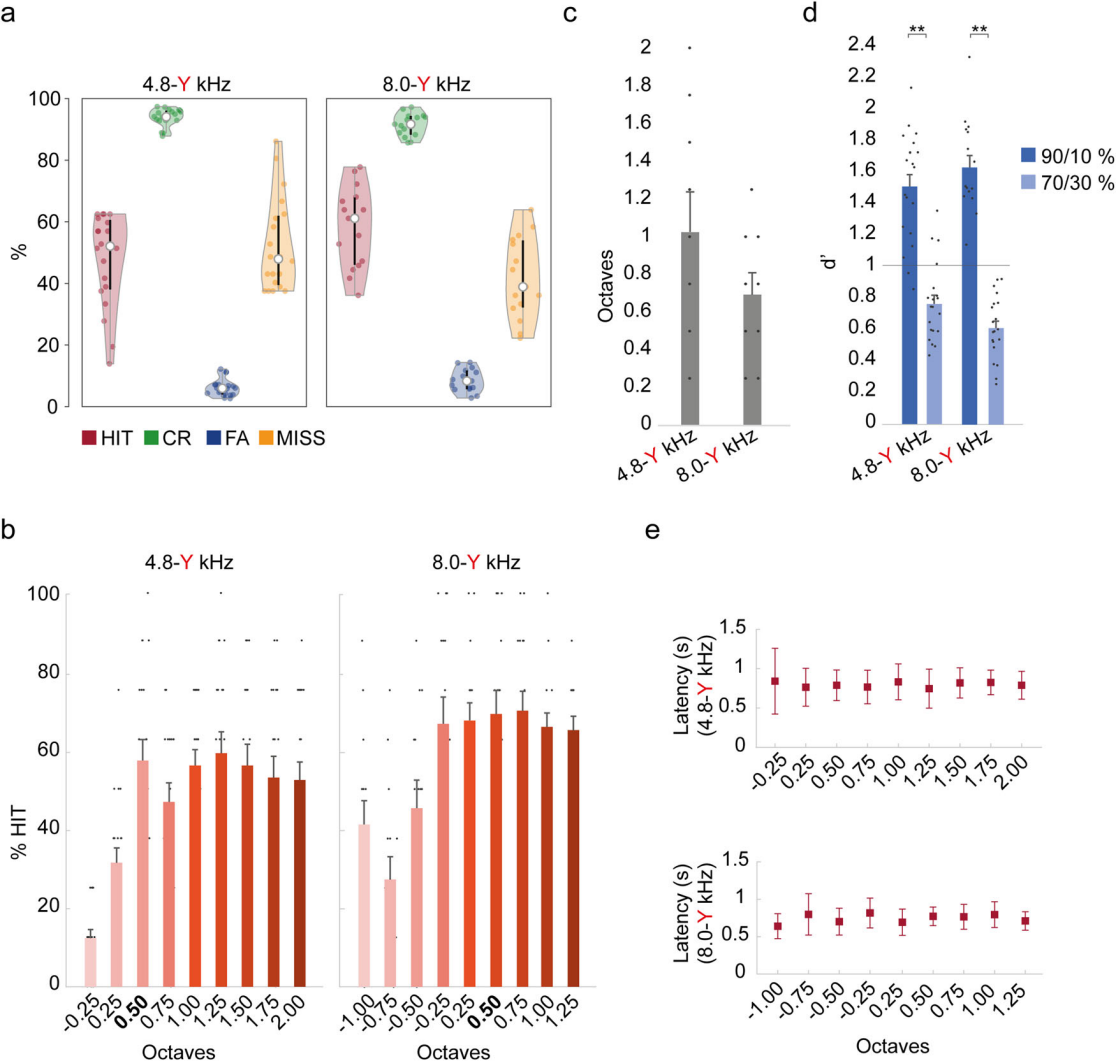
Behavioural responses to many-deviants sequence task. **a** Violin plots showing the distribution of averaged percentage (across 5 sessions/animal) of hits (HIT, in red), correct rejection (CR, in green), false alarms (FA, in blue) and missed responses after a deviant tone (MISS, in yellow) performed by trained animals distributed in different groups (4 rats/group) made by the pair of standard-deviant tone frequencies used during training (4.8–6.7 kHz, 8.0–11.3 kHz). In this paradigm, every deviant tone varies randomly in frequency from 9 possibilities (4.0, 4.8, 5.7, 6.7, 8.0, 9.5, 11.3, 13.5, 16.0 and 19.0 kHz) while the standard frequency is unchanged from the training (excluded from the deviant possibilities), the ISI was 1.5 s and the Std./Dev. probability was 90/10%. The thick black bars expand from the first (Q1) to the third quartile (Q3), and the whiskers show the range of lower and higher adjacent values. **b** Bar chart showing the percentage of HITs for each frequency contrast presented (mean ± S.E.M. of all animals per group) in the many-deviant task. **c** Bar chart representation of the average frequency contrast ± S.E.M. between the standard tones in trained groups. **d** Bar chart showing *d’* values (mean ± S.E.M. of all animals per group) obtained after the many-deviant task for both standard/deviant probabilities tested (90/10, 70/30%; two-way ANOVA test for repeated measures, *P* < 0.05). Note that for *d’* values of trained animals were *d’* > 1, the set discrimination threshold stablished (dashed line). Asterisks (*) represent significant differences between the two probabilities for each group (*p < 0.05, **p < 0.01, ***p < 0.001; three-way ANOVA test for repeated measures, *p* < 0.05). **e** Plot representing the mean latency of res*p*onse for the HIT for each frequency contrast presented (mean ± S.E.M. of all animals per group) in the many-deviant task (one-way ANOVA test, n.s.).

The last 5 sessions consisted of one final modification to the auditory discrimination task. In this case, the stimulus sequence consisted of an oddball paradigm where the frequency contrast between standard and deviant frequencies was randomly varied from 9 possibilities while the standard tone remained constant with an ISI of 1.5 s (Fig. 2c). The purpose of this test was to confirm that rats were responding to any deviant tone that violated the regularity established by the standard tone. As in previous settings, all trained rats completed the task with a high percentage of HIT (52.3 ± 2.5%) and CR (92.9 ± 0.6%) responses (47.9 ± 3.4%/93.9 ± 0.6% and 58.2 ± 3.4%/91.5 ± 1.0% for groups 4.8-Y kHz and 8.0-Y kHz). Results were compared with two-way ANOVA tests for repeated measures for %HIT and %CR, with Group and Session as factors (*p* > 0.05; Fig. 5a and Supplementary Table 8) and showed that rats generalized the deviant, *CS*+ tone to any frequency that deviates from the sequence of standard sounds that are regularly repeated. In general, the response rate of the animals for each deviant presented increased as the frequency contrast increased, showing a similar trend to that observed in the paradigms of the previous task (Fig. 5b). We should note that negative frequency deviants performance is comparatively worse, suggesting that animals prefer higher frequency deviants. This is consistent with previous works showing that neuronal SSA is higher at the high frequency edge of the frequency response areas^18,20^. In the case of the group 4.8-Y kHz from the 0.5 octaves contrast the HIT rate starts to increase and remains constant for the rest of the larger contrasts presented, showing significant differences when we evaluated the effect of the contrast presented, (three-way ANOVA test for repeated measures with Group, Session and Deviant as factors, F_Deviant_ (8,24) = 12.242, *p* < 0.001). While in the 8.0-Y kHz group it is from the −0.25 octaves contrast when the percentage of HITs remains constant with slight variations, but it occurs the same as in the other group with the statistics (three-way ANOVA test for repeated measures, F_Deviant_ (8,16) = 20.648, *p* < 0.001). Interestingly, for the negative octave separations (like −0.5 and −0.25) results show a HIT rate higher than for larger negative octave separations (for the 8 kHz group). Note that for the group of 8.0-Y kHz the octave difference is larger than for the group 4.8-Y kHz (Fig. 5c). The *d’* values were above 1 for all animals (Fig. 5d), the group 4.8-Y kHz showed a *d´*= 1.5 ± 0.1 while in the group 8.0-Y kHz the *d’* was 1.6 ± 0.1 (two-way ANOVA test for repeated measures with Group and Session as factors, *p* > 0.05; Fig. 5d and Supplementary Table 8) both similar to the results obtained in the other tasks with an ISI of 1.5 s and a probability of 90/10% (1.5 ± 0.1, 1.6 ± 0.1, 1.9 ± 0.1 and 1.9 ± 0.1 for 0.50, 0.75, 1.00 and 1.25 octaves, showing significant differences just with the paradigm with a contrast of 1.25 octaves, *p* = 0.030; three-way ANOVA test for repeated measures with Sequence, Group and Session as factors, F_Sequence_ (4,20) = 6.041, *p* = 0.002; Supplementary Table 8). We also tested the response latency of the HIT responses for each deviant tone presented from the 9 possibilities, to detect whether the reaction time is lower when the presented deviant is the training tone for each group. The HIT latency remains constant for all the deviants presented with a mean of 0.80 ± 0.05 s for the group 4.8-Y kHz and 0.74 ± 0.05 s for the group 8.0-Y kHz and show no differences with the latencies for the other paradigms presented (one-way ANOVA test; Holm–Sidak method for multiple comparisons, n.s.; Fig. 5e) and 0.65 ± 0.02 s and 0.56 ± 0.01 s for the FA latency (groups 4.8-Y kHz and 8.0-Y kHz, respectively).

To test how the standard/deviant probability affects the ability of the rats to discriminate deviant, *CS*+ tones we presented the many-deviant task changing the probability from 90/10% to 70/30%. As in previous tasks, results were obtained after 5 consecutive sessions. The performance in both trained groups was affected by this manipulation showing a reduction in the percentage of hits and correct rejections in the high deviant probability of 30% compared to the 10%. The mean ± SEM percentage values for HIT and CR responses for all rats in all trained groups were 35.1 ± 1.2% and 85.4 ± 0.9%, respectively (two-way ANOVA tests for repeated measures of %HIT, %CR with Session and Group as factors; Supplementary Table 9). When the responses of these trained animals were computed as *d’* values, they were all below 1 (Fig. 5d), and significantly lower than with the probability of 90/10% (three-way ANOVA test for repeated measures of *d’* with Probability, Session and Group as factors, F_Session_ (4,20) = 1.316, *p* = 0.298; F_Group_ (1,5) = 0.039, *p* = 0.852; F_probability_ (1,5) = 33.180, *p* = 0.002; Fig. 5d). Under this probability conditions the *d’* from the group 4.8-Y kHz was *d’* = 0.8 ± 0.1 higher than the *d’* from group 8.0-Y kHz (*d’* = 0.6 ± 0.04; two-way ANOVA test for repeated measures with Session and Group as factors, *p* > 0.05; Fig. 5d and Supplementary Table 9).

### HIT responses dependence on the number of previous standard tones

**Fig. 6:**
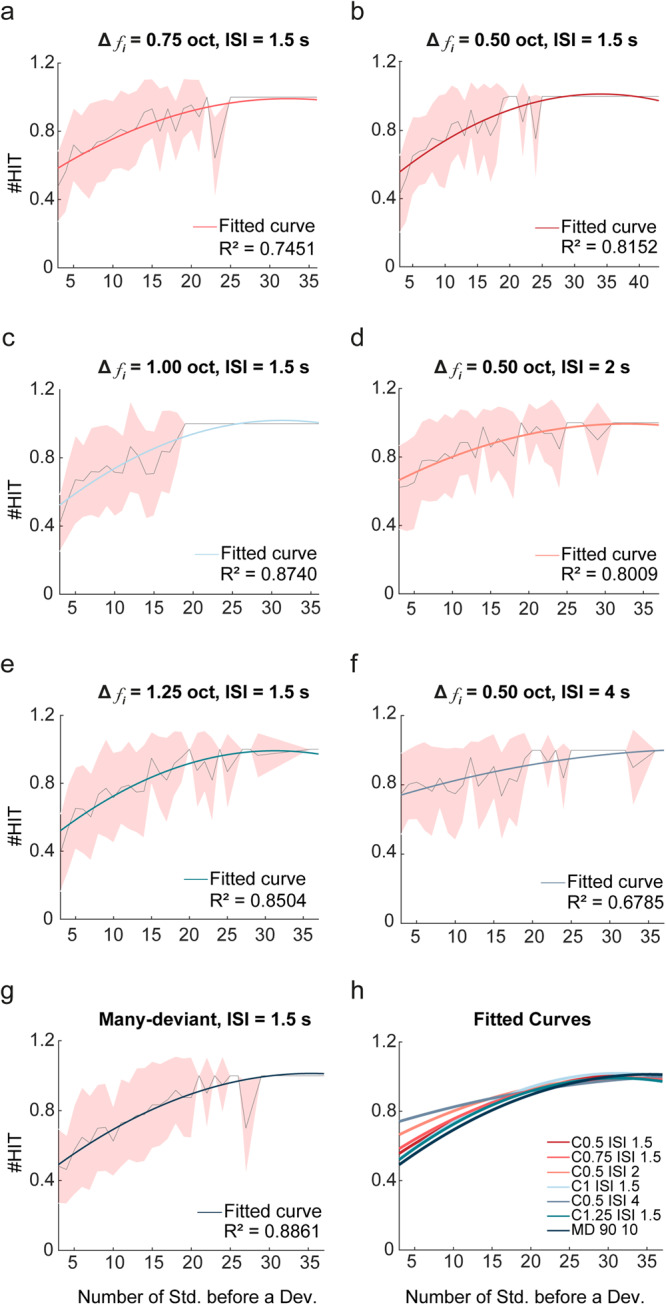
HIT responses depending on the number of standard tones between two deviants. Graphs representing the rate of HIT responses depending on the number of standard tones before a deviant of all the animals trained across the different sessions of each paradigm tested with a standard/deviant probability of 90/10%. The thin grey line shows the normalised mean of HIT responses recorded with a given number of standard tones between two deviants. The error is shown as the red shaded area, which was calculated using the standard deviation. The thicker coloured line is a second-degree polynomial curve fit. **a**, **c**, **e** Graphs obtained when presenting an oddball paradigm where the ISI was maintained as 1.5 s and the frequency contrast varied between three different options (0.75, 1.00 or 1.25 octaves). **b**, **d**, **f** Graphs obtained when presenting an oddball paradigm where the frequency contrast was maintained as 0.50 octaves and the interstimulus interval varied between three different time periods (1.5, 2 or 4 s). **g** Graphs obtained when presenting the many-deviant sequence with 9 different frequencies. **h** Representation of all the fitted curves in the same graph. Note the similarity between all the responses to each of the paradigms presented.

To assess whether the degree of adaptation to the standard tone is related to the ability of the rats to detect a deviant tone, we evaluated the number of HITs as a function of the number of standard tones prior a deviant tone (Fig. 6; normalised average response of all the sessions performed at a given frequency contrast and ISI; tendency curves were fitted to a second-degree polynomial curve). The minimum number of standard tones between two deviant tones was 3, since that was a set value when creating the oddball or many-deviant sequence. The number of HITs of each animal across the different sessions and paradigms presented show a similar pattern, with a distinct increase in HIT responses as the number of standard tones between two deviants increases. There is also a plateau around 25 standard tones between deviants, which suggests that as the number of *CS*− increases, the responses to the *CS*+ is also more robust likely due to an expectation enhancement effect.

### Evaluation of the behavioural responses over time

**Fig. 7:**
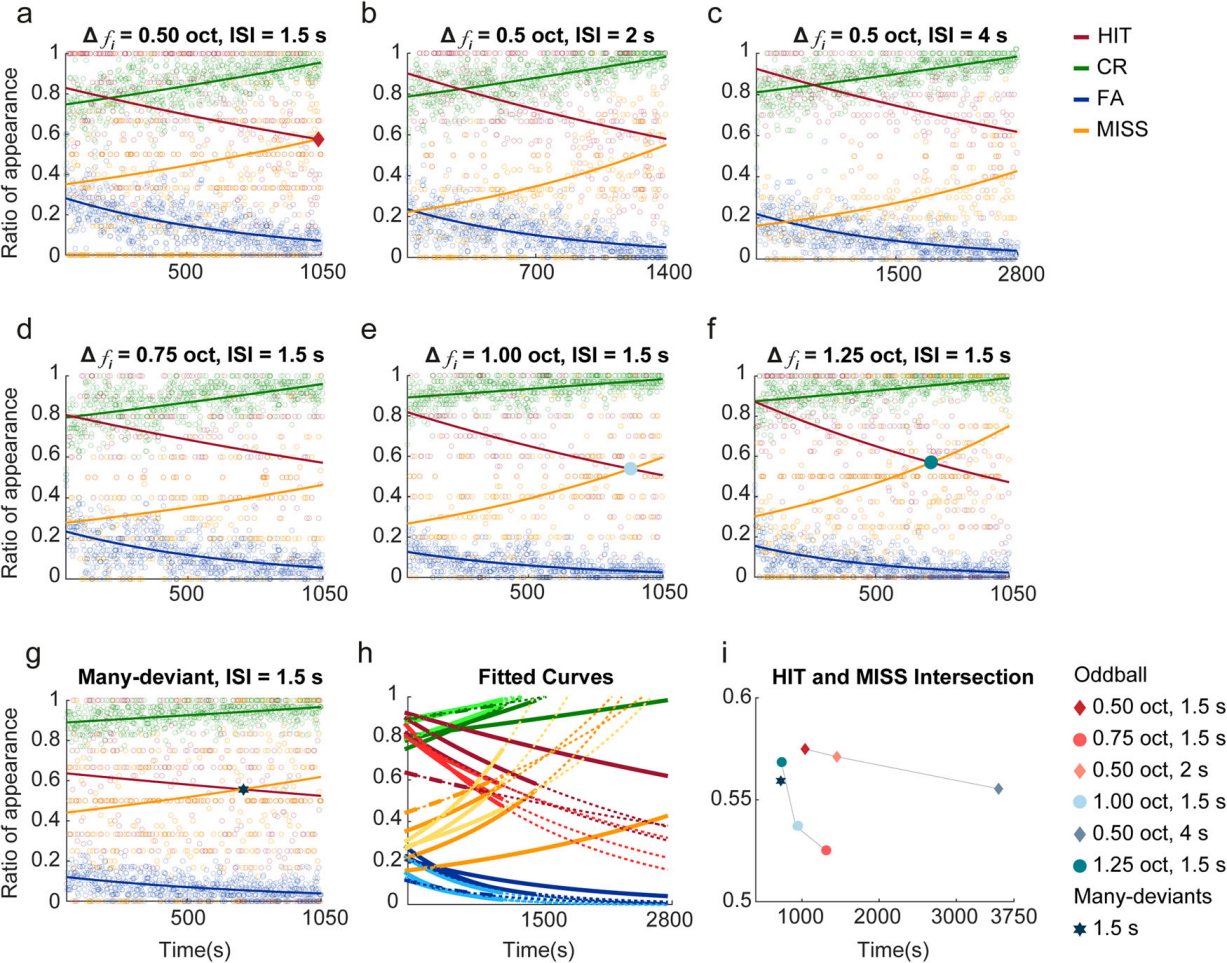
Behavioural responses over time. Time scatter plots showing the normalised behavioural responses (HIT, CR, FA and MISS) of the average of all the sessions that were performed using the same frequency contrast (C) (0.50, 0.75, 1.00 or 1.25 octaves) and interstimulus intervals (ISI) (1.5, 2 or 4 s), all of them under a standard/deviant probability of 90/10%. The tendency curves of each response are shown in the corresponding colour (red, green, blue and yellow, respectively). **a**–**c** Representation of the average response to an oddball paradigm with a frequency contrast of 0.50 octaves and an ISI of 1.5, 2 or 4 s, respectively. **d**–**f** Representation of the average response to an oddball paradigm with an ISI of 1.50 s and a frequency contrast of 0.75, 1.00 or 1.25 octaves, respectively. **g** Average responses to the many-deviant task with an ISI of 1.5 s. **h** Representation of all fitted curves, with darker colours for the different ISIs with a contrast of 0.5 octaves and lighter colours for the different contrasts tested under an ISI 1.5 s. The many-deviant task is represented with a different pattern. The discontinuous line is the tendency the fitted curve has. **i** Plot showing the moment of intersection between HIT and MISS for each of the paradigms presented.

Finally, to evaluate the engagement of the animals in the different tasks throughout the testing sessions, we plotted the behavioural responses over time, and normalised them to the number of presentations of standard or deviant tones, respectively, across all sessions (Fig. 7; tendency curves were calculated using a single term exponential model).

These graphs show that, irrespective of the ISI and the frequency contrast used, there is a distinct decrease in FA and HIT responses, and an increase in CR and MISS responses over time (Fig. 7). Interestingly, when we examine how the HIT and MISS responses interact, we observed a clear tendency, such that this interaction occurs as a function of time and is dependent on the frequency contrast and ISI tested. When this interaction is analysed for the ISI, we see that it occurs later in time as the ISI increases, while the opposite occurs as the contrast between standard and deviant tones increases.

## Discussion

We employed the widely used oddball paradigm in single-unit recordings and adapt it to a behaviour discrimination task. Hence, we trained freely moving rats to discriminate auditory stimuli under various modifications of the classic oddball paradigm.

Our experiments demonstrate that all animals successfully detected a deviant tone (*CS*+) embedded in a series of repetitive standard tones. Interestingly, although animals were overtrained with a specific deviant and standard tone, constant across shaping protocols, the rats were able to perform the task when further tested with other deviant tones higher in frequency than the original. This set of behavioural tests will be useful for future experiments that will try to determine whether such detection is dependent on phenomena such as SSA.

While there are dozens of examples in the literature of behavioural studies in the auditory domain that use auditory discrimination tasks (e.g., refs. ^41,45–50^), to date, there are few reports that specifically use an oddball sequence in a discrimination task^31,51,52^ especially for enabling context-dependent behaviour^53^. Psychophysical data from monkeys trained to detect increments in frequency, showed a substantial improvement on the task^45^. Similarly, trained freely-moving rats also demonstrated a direct relationship between the HIT rate and stimuli contrast^46^. Although small differences were observed between the individual groups depending on the frequencies used during training in our experiments, there was a direct relationship between the *d’* and the frequency contrast used. A previous study^54^ has induced generalization in the detection of a conditioned tone from another unconditioned tone, less than an octave apart. Thus, de Hoz and Nelken’s results support the notion that our results are likely due to a frequency contrast effect.

An important feature that characterizes SSA is its dependency on various stimulus parameters, including frequency separation, probability of occurrence of the deviant tone, and/or stimulation rate (time interval between stimuli). These SSA sensitivities have been reported across different auditory regions in multiple studies^10–24^.

The manipulation of the probability of occurrence of the deviant stimuli has an important impact on the magnitude of observed SSA^11,27^. Different deviant probabilities, specifically 30% and 10%, have been evaluated, revealing that SSA exhibits an upward trend as the deviant probability decreases^10,11^. This is consistent with our results since all trained animals show a reduction in the response rate to the deviant tones when the probability tested was 30% rather than 10%. Furthermore, the detection of deviant tones depends on the number of preceding standard tones, which leads to the induction of adaptation. Based on this feature, we evaluated the degree of adaptation to the standard stimulus as a function of the number of presentations. Our results show that an animal’s response to the deviant tones increases as it is preceded by a larger number of standard sounds. So, as it occurs at the neuronal level, from a behavioural perspective, each animal’s discrimination ability is sensitive to stimulus probability, with greater sensitivity to tones that are less likely to occur^11^. These findings also support that there might be some sort of transitional probability occurring at the behavioural level.

Memory trace refers to the shortest inter-stimulus interval (ISI) where the response to a stimulus remains unaffected by its preceding stimulus^5^. Studies have shown that SSA in the auditory cortex or thalamus tends to increase^10^ at ISIs shorter than 2 s^17,55^. Our animals show a good performance for the three ISIs tested, but the best results were obtained with the lowest stimulation rate (ISI of 4 s). Interestingly, if SSA is indeed the main mechanism underlying the described behavioural results, we would have expected that the deviant detection improves with decreasing repetition rate because neuronal SSA indices typically increase with decreasing ISIs up to a certain point^10,11,17,55^. More notably, we observed that the animals´ engagement lasted longer doing the task than when testing shorter ISIs. This finding suggests that longer time intervals between tones allow the animals to recover from the previous stimulation and that this longer period allows them to sustain the attention for extended epochs, which leads to them performing improved deviant discrimination.

To assess the animals’ engagement with the task, we evaluated the interaction between their different responses throughout the session and we observed a consistent pattern across all paradigms. There is a gradual change where the animals’ active responses (HIT and FA) decline, while the occurrence of correct rejections and miss responses increases. This observation suggests that the animals lose their attention to the task at a particular point during the session. However, the time course over which this occurs varies depending on the specific task presented. Notably, when longer ISIs are tested, the animals maintain their attention for a longer period of time. This phenomenon can potentially be attributed to the animals having more time to recover between stimuli. In the context of the many-deviant task, this instance occurs earlier in the session probably because of the increased difficulty of this task, which demands a higher level of cognitive effort from the animals, and suggests an earlier onset of task fatigue. Alternatively, the reward consumed during the protocol is an important factor in explaining the decrease in the number of active responses. In this context, in operant conditioning, the decision of the rat to access the pellet is mediated by their inclination to eat. Thus, this satiety could be an important factor in influencing operant performance and the number of active responses made^56^.

We designed the many-deviant behavioural task paradigm that allowed us to manipulate a physical feature of the sound, i.e., frequency, as a simple way to test the regularity established by the standard tones in the sound presentation pattern. This is because several deviant tones are embedded in the sound sequence, not just one deviant, as it occurs with the classical oddball paradigm where only two tones are used: one being the deviant and the other the standard. The implementation of this paradigm confirmed that animals responded to any violation of the sound pattern regularity^57^, regardless of the sound frequency, making the detection of this violation context-dependent and not simply a memory or learning process specific to a particular deviant tone. Future studies should test other aspects of the regularity in the many-deviant sequence, such as intensity, sound duration, or the probability of the deviants to generalize this finding. Polley and colleagues^58^ designed a task that might be a reverse to our many-deviant sequence. Trained rats had to respond to a specific tone target embedded in a cloud of distractors. Under this paradigm, animals showed a reduction in their discrimination ability as a function of the number of distracting tones^58^. Although the experimental design in Polley’s study is different to ours, their results agree with our data, because discriminability decreased as the number of deviant tones increased. Furthermore, our many-deviant task implements an additional advantage, as the animals not only need to detect a tone (tone detection task) but they also need to set a threshold in order to separate a given tone from the others (tone discrimination task). The many-deviant task also suggests that rats have the ability to detect genuinely novel stimuli because each deviant stimulus is new and different from the standard and the previous deviants. Local field potential (LFP) recordings in A1 using head-fixed rats performing a two-alternative forced-choice task have associated selective learning and SSA^32^. Their results show better performance occurring as the frequency contrast increases between the target tone (homologous to our deviants) and distractor tones (homologous to our standards), which further supports our results. A reduction in response to the target frequency was justified through SSA, as better discrimination occurred when the distractor frequency was adapted. Another study, also based on LFPs, in conditioned awake freely moving rats performing auditory discrimination tasks^33^, showed that the contrast between standard and deviant responses remained unchanged or decreased for conditioned tones but increased for unconditioned ones. However, deviant stimuli (pure tones or complex sounds) were aversively conditioned and the tested SSA used sounds that have not been introduced during conditioning. Thus, direct comparisons with our experiments are limited in value.

While we have not recorded neuronal SSA responses during the behavioural tasks, it is tempting to speculate that the similarity of the present results and those reported on the response properties of neuronal SSA at cortical and subcortical levels^11,17,18,20,30^ are consistent and congruent with behavioural correlate of neuronal SSA. Currently, SSA is best explained under the predictive coding framework^12^. According to this conceptual paradigm^59,60^ the increase in SSA may be due to an increased response to deviant sounds and/or an increase of adaptation to repeated sounds^12^. An enhancement in the deviant responses is referred to as genuine deviance detection or prediction errors. Prediction error signals are weighted by the precision of the prediction^61^, and more precise prediction errors elicit larger responses. This is probably the mechanism through which attention operates. In other words, attention modulates the prediction error signal adjusting its gain, and paying attention to a stimulus facilitates increased precision. Thus, more attention = increased precision = increased gain = larger prediction error. Several studies have shown that attention can counteract^59^ or even reverse^62^ the suppressive effects of sensory adaptation. There is currently an avid debate regarding the definitions of attention and prediction, since these two concepts have been mixed previously, and a proposal which is currently well accepted is to conceptualize attention exclusively as task-relevance^63^. But this is still a matter of debate that awaits future studies for clarification. Thus, after training, sounds have become task-relevant, which means that the animals pay more attention to them, and therefore, the prediction errors should be larger. This is precisely what we observed. However, it should also be considered that after learning, the task may even become easier and the animals have to pay less attention^64^.

In the analysis of the ABRs, we found that the response latency decreased after the training period. This is consistent with the theory that training induces task-relevance. This leads to animals developing a plasticity to the auditory stimulus, not only to the frequencies of the behavioural tasks, so that they are therefore able to detect any of these tones presented more swiftly. Previous studies have shown that training animals with an augmented acoustic environment develops some form of neuronal plasticity, not only during development and critical periods^65,66^ but even during adulthood^67^. Modelling studies suggest that individual neurons can switch their responsiveness between various input signals by adjustment of excitatory–inhibitory balance (e.g., refs. ^68,69^) and similar mechanisms may play a role at the neuronal level to develop plasticity and enhance behavioural performance as we show in our experiments. But this conceptualization awaits experimental confirmation at the neuronal level.

The mechanisms of auditory habituation at the cortical and subcortical levels have received relatively little attention^69^, but a recent study, using two-photon calcium imaging, has demonstrated habituation in the AI of mice^70^. These authors discovered that responses of layer 2/3 pyramidal neurons decreased after daily periods of repetitive stimulation and that the decrease was associated with an up regulation in the activity of somatostatin-expressing inhibitory neurons. These changes take place over a much longer time-scale than most forms of SSA, but the relationship between the two effects of repetitive stimulation and the mechanisms that produce those effects are clearly related^49^. Thus, the improvement in discriminative ability as a consequence of training as we demonstrate here, is one of the forms of sensory system plasticity that has driven profound changes in our conceptualization of sensory function and perceptual learning^49^.

In summary, we have addressed an important and critical question which potentially has major impact not only for understanding apparently simple behaviour and neurophysiological habituation but more generally the mechanisms underlying prediction errors, which are extensively studied in humans and animal models.

## Methods

All experimental procedures were carried out at the University of Salamanca and all methodological procedures were approved by the Bioethics Committee for Animal Care of the University of Salamanca (USAL-ID-574 and 683) and performed in compliance with the standards of the European Convention ETS 123, the European Union Directive 2010/63/EU, and the Spanish Royal Decree 53/2013 for the use of animals in scientific research.

We conducted experiments on 8 female Long Evans rats (Janvier-Labs, Le Genest-Saint-Isle, France), aged 7–10 weeks old with body weights between 200–250 g. Animals were housed at an animal care facility in the Institute for Neuroscience of Castilla y León. Temperature and humidity were controlled and maintained on a 12 h light/dark cycle, with free access to water and standard rodent food pellets until the beginning of the experiment. Once the experiment began, animals were kept on a restricted feeding schedule to maintain their body weight at 90–95% of the initial weight. Rats were weighed and fed daily under those constraints.

### Auditory brainstem response (ABR) test

We induced deep anaesthesia using a mix of ketamine (50 mg/kg, i.m.; Imalgene) and dexmedetomidine (0.25 mg/kg, i.m.; Sedadex). We then recorded auditory brainstem responses (ABR test; Fig. 1e) using subcutaneous electrodes to ensure that the experimental animals had normal hearing in both ears. Following standard procedures as previously done in our lab^21^, and elsewhere^44^. ABR stimuli consisted of 100 µs clicks at a 21/s rate, delivered monaurally in 10 dB steps, from 10 to 90 decibels of sound pressure level (dB SPL), using a closed-field speaker.

ABR responses were collected using a RX6 multifunction Processor (RZ6 Multi I/O Processor; Tucker-Davis Technologies, TDT) and processed with BioSig software (Tucker-Davis Technologies). An anaesthetic reversal agent (1 mg/kg, i.p.; Atipamezol) was given after ABR tests to recover animals. Thereafter, animals were kept in their cage with freely available food and water for at least 3 days prior to behavioural experiments.

### Behavioural apparatus

All behavioural experiments were carried out in a Med Associates operational cage (30 × 25 × 19 cm modular chamber with a grid floor, mod. ENV-008, Med Associates, Inc., Georgia, USA), controlled by custom-made scripts (written in Trans V software by Cibertec, S.A., Madrid, Spain) with a smart interface controller (DIG-716P2). The operational cage was equipped with a house light (ENV-215M), that was turned off before the testing session began and turned on at the end of every session. The light was located on the top-central part of the left panel in the cage. The speaker (ENV-224BM) was positioned on the top and rear part of the left panel and was controlled by a sound generator (ANL-926). Calibration of the speaker was made using a ¼-inch condenser microphone (model 4136, Brüel & Kjaer) and a dynamic signal analyser (Photon+, Brüel & Kjaer). A single nose poke port (ENV-114BM) was installed adjacent to a food tray for reward delivery in the right panel of the cage. Rewards consisted of 45 mg dustless precision pellets (Bilaney Consultants, Düsseldorf, Germany), dispensed by a modular pellet dispenser (ENV-203M) connected to the food tray for rewards (Fig. 1c). These responses were automatically quantified by software MED-PC V version 5.1 (Med Associates, Inc.). All sessions were recorded using a HD LED IR camera (ELP Ailipu Technology Co., Ltd, Shenzhen, Guangdong, China) placed to capture a central view.

### Shaping protocol

To evaluate the ability of the rats (*n* = 8) to detect and discriminate a sound embedded in each auditory environment, we developed a behavioural go/no-go operant conditioning procedure based on an auditory discrimination task and consisting of 5 consecutive stages.

*Stage 1* (7 sessions): Rats were habituated to handling (twice per day) for 1 week (Fig. 1a). Stage 1 consisted of 30 min of habituation to the experimental room, followed by 30 min of handling. Mean weight during over 3 days was established as the initial weight (measured at the end of the day). At this point, we initiated food restriction of the animals by removing food entirely, and only feeding them once per day, so that their weight was maintained between 90–95% of the initial weight until the end of experiment.

*Stage 2* (3 sessions): Following Stage 1, rats were placed into the operant chamber. To overcome neophobia, 5 reward pellets were placed in the cage at the beginning of the session, and 3 unexpected pure tones were randomly presented using the overhead speaker (70 dB, duration 400 ms, rise/fall 10 ms). Two groups of 4 animals were established (Supplementary Table 1) according to the tone used in this stage of training. The frequency used in this stage corresponded to the one used as a deviant tone in the next stage during the oddball paradigm (see below).

*Stage 3* (3–5 sessions): Next, the rats were required to learn the association between the execution of a nose-poke response after a cue tone (the same tone used in Stage 2) and reward delivery (*CS*; Fig. 1a, Nose-poke shaping). Once the animal was placed in the cage, the programme would initiate after 1 min, and the first tone was presented 1 s later. When the rat made a nose-poke response, one pellet was delivered. There was no limit to the time following a tone to facilitate this association. The association between the nose-poke and the reward delivery was being learnt in this phase, and the tone was played 1 s after each nose-poke stimulation. Animals took approximately 3 sessions to get the maximum number of rewards (15) in less than 30 min.

*Stage 4* (9 sessions): Next, a limited hold was applied, so that rats needed to nose-poke within a specific time of *CS* (the same tone used in Stage 2) presentation. The *CS* was presented every 2.5 s. Initially, the limited hold was 2.5 s (Fig. 1a, Response time shaping). In the following sessions, the limited hold was progressively reduced to 1.5 s and the interval between *CS* presentations was increased to 4.5 s.

*Stage 5* (10–15 sessions): In the last stage of the training protocol, a classical auditory oddball paradigm (Fig. 2a) was presented (Fig. 1a, Oddball training;), consisting of a rare deviant tone (*DEV*, 10%), which was the same tone used as the *CS*+ in previous stages, and a regular standard (*STD*, 90%) with a different frequency to DEV, which was not associated with rewards (*CS*−). The auditory stimuli were presented once every 1.5 s, and only nose-pokes within a limited hold (response window) of 1.49 s after the DEV (*CS*+) were rewarded. Possible responses (Fig. 1c) were quantified as Hits (nose-poke responses in the limited hold after a deviant tone, rewarded with one pellet; HIT), False alarms (responses to a standard tone; FA), Correct rejections (absence of response after a standard tone; CR) and Missed responses (absence of response to deviant tones; MISS). Each FA was punished with 5 s time-out. Rats completed training when they reached criterion performance (*d’* ≥ 1; see below) for 3 consecutive sessions (Fig. 1d).

### Behavioural protocols

Different behavioural protocols were generated to confirm the animal’s ability to discriminate and detect pure tones.

#### Oddball sequence task (15 sessions)

To receive food rewards, animals were required to respond with a nose-poke to the occurrence of a low probability deviant tone (*CS*+, 10%) in a sequence of high probability standard tones (*CS−*, 90%). The frequency contrast between tones was set at 0.5 octaves (Fig. 2a). Each pure tone (70 dB, duration 200 ms, rise-fall 10 ms) were spaced by an interstimulus interval of 1.5 (onset to onset; ISI), 2 and 4 s for five consecutive sessions each; while the response window was maintained with a duration of 1.49 s for the three ISIs tested. Supplementary Table 1 shows the frequencies used for each group. The maximum number of standard (630) and deviant (70) tones were calculated for a session of 20–40 min, depending on the ISI applied (700 tones). The first 5 stimuli of each session were standard tones, and each deviant tone was always preceded by a minimum of 3 standard tones. We acquired data during 5 consecutive sessions for each ISI, applying these sequences to every animal. Responses were quantified using *d’* values.

#### Deviant frequency contrast variation task (30 sessions)

As in the previous task, to receive food rewards, animals were required to respond to the low probability deviant tone (10%) in a sequence of high probability (90%) standard tones, but in this case, the frequency contrast was modified. Standard frequency was fixed across sessions, and the frequency of the deviant tone was 0.75, 1.00 and 1.25 octaves larger than the standard frequency (Fig. 2b). We acquired data during 5 consecutive sessions for each frequency contrast tested, applying these sequences to every animal. Responses were quantified using *d’* values. We also tested the previous frequency contrasts (0.75, 1.00 and 1.25 octaves) for another fifteen sessions, but through these sessions, we changed the probability of the deviant tone to 30% and the probability of the standard tone to 70%. The maximum number of standard (493) and deviant (207) tones were calculated for a session of 30 min (700 tones). Responses were quantified using *d’* values.

#### Many-deviant task (10 sessions)

To receive food rewards, animals were required to respond to different deviant tones (10%) presented in a sequence of a constant high probability (90%) standard tone. Each deviant tone was a randomly selected frequency from 9 possibilities (4.0, 4.8, 5.7, 6.7, 8.0, 9.5, 11.3, 13.5, 16.0 and 19.0 kHz, excluding the standard frequency for each group). This results in a 1.11% probability of occurrence for each different deviant. To maintain this probability, the maximum number of standard (648) and deviant (72) tones were calculated for a session of 20 min (720 tones). The standard tone frequency remained unchanged during the task (4.8 or 8.0 kHz; Fig. 2c and Supplementary Table 1). For the last five sessions, the probability of the deviant tone was changed from 10% to 30%.

First, the animals performed the deviant frequency contrast variation task with a STD/DEV probability of 70/30%, increasing the frequency of the deviant tone by 0.25 octaves every 5 sessions, then the many-deviant task also with the 70/30% probability. Secondly, the animals were presented the STD/DEV probability tasks of 90/10%, the order of presentation was performed in a quasi-randomized way for both groups (Supplementary Table 10). After the last session was completed, animals were returned to *ad libitum* food access for 3 days and a final weight was measured.

### Behavioural analysis

To determine the animal’s ability to recognize deviant tones intermingled in the paradigms presented, we calculated the so-called *d’* discrimination index (adapted from Green and Swets^71^):

1 $${d}^{{\prime} }={f}_{1}\left(x,\mu ,\sigma \right)-{f}_{2}(y,\mu ,\sigma )$$

where *f* is the normal distribution of the probability of *x* (HIT) or *y* (FA), *µ* is the mean of the distribution and $\sigma$ is the standard deviation. In cases with a minimum (0) or a maximum (70) of HIT or FA responses, it was not possible to calculate *d’*, and application of Hautus’ correction was necessary. The correction consisted of adding 0.5 to both the number of HIT and FA responses, and adding 1 to both the number of deviants and the number of standards: dubbed the *log-linear approach*^72^. For the many-deviant task *d’* was quantified for all deviant tones pooled together, regardless of the frequency presented.

All figures were obtained using MATLAB R2022A. The tendency curves for the HIT response analysis graphs were calculated using a second-degree polynomial curve fit, where the goodness of the fit was obtained by calculating R-Square, which shows how successful the fit is adjusting to the variation of the data. The thin grey line (Fig. 6) was normalised by dividing the number of times a HIT was performed by the times a deviant tone was presented at a set amount of standard tones between two deviants. The tendency curves for the behavioural responses over time graphs (Fig. 7) were calculated using a single term exponential model.

### Statistics and reproducibility

A total number of 8 rats were conducted in this study. Animals were randomly assigned to 2 different groups (4 rats each). For each group, we used different pairs of sound frequencies, but the standard tone was kept constant per group through all the sessions and the different options of the task (Supplementary Table 1).

The statistical analysis for ABR recordings was conducted using a three-way ANOVA test for repeated measures evaluating the wave amplitudes and the latency of the responses [left and right ears (first factor) obtained before and after (second factor) the experimental procedures with the different groups of rats (third factor)] using SPSS v.28 (IBM, Armonk, NY, United States; Supplementary Table 2).

The statistical analysis of the data derived from behaviour tasks (averages of 5 consecutive sessions) was performed using SPSS v.28. Comparisons of all responses recorded (%HIT, %CR, %FA and %MISS), as well as calculated *d’* values, were assessed by two- or three-way ANOVAs for repeated measures with factors: group of rats, session and the different variables applied in each paradigm (ISI and frequency contrast). As *post-hoc* comparisons, we used the Holm-Sidak method (Supplementary Tables 2–9).

### Reporting summary

Further information on research design is available in the Nature Portfolio Reporting Summary linked to this article.

### Supplementary information

Supplementary Materials

Description of Additional Supplementary Files

Supplementary Data 1

Reporting Summary

## Data Availability

The datasets generated during the current study are available from the corresponding author on reasonable request and at GREDOS repository from the Salamanca University with the following identifier: http://hdl.handle.net/10366/153079. The source data for the graphs and charts in the manuscript can be found in Supplementary Data 1.
