# Supplementary material for: Novelty detection in an auditory oddball task on freely moving rats
Source: Commun Biol. 2023 Oct 19;6:1063. doi: 10.1038/s42003-023-05403-y (PMC10587131; doi:10.1038/s42003-023-05403-y)
Supplement: Supplementary file 1 — Supplementary Materials [file 42003_2023_5403_MOESM1_ESM.pdf]

Supplementary Table 1		
**GROUPS**	**TRAINED FREQUENCIES (kHz)**	**NUMBER OR ANIMALS**
1	4.8 – 6.7	4
2	8.0 – 13.0	4

Experimental groups established as a function of tone frequencies used for the training and the number of animals assigned to each group.

**Supplementary Table 2**						
**Wave amplitude**						
**Group**	F(1,5) = 0.869, p = 0.394					
**Time (Before/After)**	F(1,5) = 2.503, p = 0.174					
**Ear (Right/Left)**	F(1,5) = 8.198, p = **0.035**					
**Group x Time**	F(1,5) = 0.479, p = 0.520					
**Group x Ear**	F(1,5) = 0.193, p = 0.679					
**Time x Ear**	F(1,5) = 0.341, p = 0.584					
**Group x Ear x Time**	F(1,5) = 0.424 , p = 0.543					
**Response latency**						
**Group**	F(1,5) = 0.030, p = 0.870					
**Time (Before/After)**	F(1,5) = 60.044, **p = 0.001**					
**Ear (Right/Left)**	F(1,5) = 0.123, p = 0.740					
**Group x Time**	F(1,5) = 5.685, p = 0.063					
**Group x Ear**	F(1,5) = 0.058, p = 0.820					
**Time x Ear**	F(1,5) = 1.386, p = 0.292					
**Group x Ear x Time**	F(1,5) = 0.286 , p = 0.616					
**Amplitude comparisons (Holm-Sidak method)**						
Ear	*p*	*p*(I)	*p*(II)	*p*(III)	*p*(IV)	*p*(V)
Left ear x Right ear	**0.035**	**0.027**	0.330	**0.035**	**0.035**	0.688
**Latency comparisons (Holm-Sidak method)**						
Time	*p*	*p*(I)	*p*(II)	*p*(III)	*p*(IV)	*p*(V)
Before x After	**0.001**	**0.023**	**0.000**	**0.004**	**0.004**	**0.012**

Statistical details of the ABR comparisons (cf., Fig. 1d). 3-ways ANOVA test for repeated measures. Data show significant differences across categories (p > 0.05).

**Supplementary Table 3**						
**%HIT & %MISS**						
**Session**	F(4,20) = 0.329, p = 0.855					
**ISI (1.5, 2, 4 seconds)**	F(2,10) = 1.845, p = 0.208					
**Group**	F(1,5) = 0.870, p = 0.394					
**Session x Group**	F(4,20) = 1.252, p= 0.321					
**ISI x Group**	F(2,10) = 0.955, p = 0.417					
**Session x ISI**	F(8,40) = 1.940, p = 0.080					
**%CR & %FA**						
**Session**	F(4,20) = 6.601, **p = 0.001**					
**ISI (1.5, 2, 4 seconds)**	F(2,10) = 8.733, **p = 0.006**					
**Group**	F(1,5) = 0.007, p = 0.939					
**Session x Group**	F(4,20) = 0.505, p= 0.733					
**ISI x Group**	F(2,10) = 1.253, p = 0.327					
**Session x ISI**	F(8,40) = 2.131, p = 0.055					
**d'**						
**Session**	F(4,20) = 2.275, p = 0.097					
**ISI (1.5, 2, 4 seconds)**	F(2,10) = 12.057, **p = 0.002**					
**Group**	F(1,5) = 0.594, p = 0.476					
**Session x Group**	F(4,20) = 1.276, p= 0.313					
**ISI x Group**	F(2,10) = 1.456, p = 0.279					
**Session x ISI**	F(8,40) = 3.316, **p = 0.005**					
**HIT Latency**						
**Session**	F(4,20) = 0.909, p = 0.478					
**ISI (1.5, 2, 4 seconds)**	F(2,10) = 7.937, **p = 0.009**					
**Group**	F(1,5) = 4.266, p = 0.094					
**Session x Group**	F(4,20) = 3.217, **p= 0.034**					
**ISI x Group**	F(2,10) = 0.808, p = 0.473					
**Session x ISI**	F(8,40) = 2.497, **p = 0.027**					
**FA Latency**						
**Session**	F(4,20) = 1.749, p = 0.179					
**ISI (1.5, 2, 4 seconds)**	F(2,10) = 80.281, **p = 0.000**					
**Group**	F(1,5) = 0.466, p = 0.525					
**Session x Group**	F(4,20) = 0.655, p= 0.630					
**ISI x Group**	F(2,10) = 7.094, **p = 0.012**					
**Session x ISI**	F(8,40) = 4.931, **p = 0.000**					
**d' comparisons (Holm-Sidak method)**						
**Group**	*p*	*p*(ISI 1.5 s)	*p*(ISI 2 s)	*p*(ISI 4 s)		
4.8-6.7 kHz x 8.0-11.3 kHz	0.476	0.284	0.370	0.974		
**ISI**	*p*	*p*(S1)	*p*(S2)	*p*(S3)	*p*(S4)	*p*(S5)
1.5 s x 2 s	**0.046**	0.051	**0.007**	0.893	**0.026**	1.000
1.5 s x 4 s	**0.028**	0.070	0.788	**0.002**	0.256	**0.032**
2 s x 4 s	0.181	1.000	0.987	**0.021**	0.977	**0.035**

**HITlat comparisons (Holm-Sidak method)**						
**Group**	*p*	*p*(ISI 1.5 s)	*p*(ISI 2 s)	*p*(ISI 4 s)		
4.8-6.7 kHz x 8.0-11.3 kHz	0.094	0.259	0.082	0.869		
**ISI**	*p*	*p*(S1)	*p*(S2)	*p*(S3)	*p*(S4)	*p*(S5)
1.5 s x 2 s	0.983	0.063	0.988	0.997	0.617	0.913
1.5 s x 4 s	0.072	**0.011**	0.643	0.722	0.990	**0.027**
2 s x 4 s	**0.000**	0.157	0.381	0.117	0.465	0.081
**FAlat comparisons (Holm-Sidak method)**						
**Group**	*p*	*p*(ISI 1.5 s)	*p*(ISI 2 s)	*p*(ISI 4 s)		
4.8-6.7 kHz x 8.0-11.3 kHz	0.525	0.072	0.296	0.817		
**ISI**	*p*	*p*(S1)	*p*(S2)	*p*(S3)	*p*(S4)	*p*(S5)
1.5 s x 2 s	0.236	0.299	0.103	**0.034**	0.997	0.852
1.5 s x 4 s	**0.000**	**0.002**	**0.005**	0.079	1.000	**0.001**
2 s x 4 s	**0.001**	0.071	**0.033**	0.552	0.991	**0.006**

Statistical details of the oddball paradigm responses with the different ISIs tested (cf., Fig. 3). 3-ways ANOVA-test for repeated measures (one test per category). Statistically significant comparisons ($p < 0.05$) highlighted in bold.

**Supplementary Table 4**						
**Latency ISI 1.5 s**						
**Session**	F(4,20) = 4.036, **p = 0.015**					
**Response (HIT, FA)**	F(1,5) = 5.150, p = 0.072					
**Group**	F(1,5) = 4.815, p = 0.080					
**Session x Group**	F(4,20) = 0.839, p= 0.101					
**Response x Group**	F(1,5) = 0.048, p = 0.836					
**Session x Response**	F(4,20) = 1.315, p = 0.298					
**Latency ISI 2 s**						
**Session**	F(4,20) = 2.350, p = 0.089					
**Response (HIT, FA)**	F(1,5) = 12.330, **p = 0.017**					
**Group**	F(1,5) = 2.419, p = 0.181					
**Session x Group**	F(4,20) = 1.603, p= 0.212					
**Response x Group**	F(1,5) = 5.461, p = 0.067					
**Session x Response**	F(4,20) = 0.860, p = 0.505					
**Latency ISI 4 s**						
**Session**	F(4,20) = 2.825, p = 0.052					
**Response (HIT, FA)**	F(1,5) = 53.545, **p = 0.001**					
**Group**	F(1,5) = 0.000, p = 0.993					
**Session x Group**	F(4,20) = 2.059, p= 0.125					
**Response x Group**	F(1,5) = 0.552, p = 0.491					
**Session x Response**	F(4,20) = 3.387, **p = 0.029**					
**Latency ISI 1.5 s comparisons (Holm-Sidak method)**						
**Group**	*p*	*p*(HIT)	*p*(FA)			
4.8-6.7 kHz x 8.0-11.3 kHz	0.080	0.259	0.072			
**Response**	*p*	*p*(S1)	*p*(S2)	*p*(S3)	*p*(S4)	*p*(S5)
HIT X FA	0.072	0.541	**0.025**	0.080	0.146	**0.016**
**Latency ISI 2 s comparisons (Holm-Sidak method)**						
**Group**	*p*	*p*(HIT)	*p*(FA)			
4.8-6.7 kHz x 8.0-11.3 kHz	0.181	0.082	0.296			
**Response**	*p*	*p*(S1)	*p*(S2)	*p*(S3)	*p*(S4)	*p*(S5)
HIT X FA	**0.017**	**0.006**	0.343	0.622	0.418	0.143
**Latency ISI 4 s comparisons (Holm-Sidak method)**						
**Group**	*p*	*p*(HIT)	*p*(FA)			
4.8-6.7 kHz x 8.0-11.3 kHz	0.993	0.869	0.817			
**Response**	*p*	*p*(S1)	*p*(S2)	*p*(S3)	*p*(S4)	*p*(S5)
HIT X FA	**0.001**	**0.008**	0.089	0.217	**0.005**	0.108

Statistical details of the latency comparison for the oddball paradigm with the different ISIs tested (cf., Fig. 3). 3-ways ANOVA-test for repeated measures (one test per category). Statistically significant comparisons ($p < 0.05$) highlighted in bold.

7

**Supplementary Table 5**	
**%HIT & %MISS**	
**Session**	F(4,20) = 3.781, **p = 0.019**
**Contrast (0.50, 0.75, 1.00, 1.25 octaves)**	F(3,15) = 0.804, p = 0.511
**Group**	F(1,5) = 0.517, p = 0.504
**Session x Group**	F(4,20) = 2.823, p= 0.052
**Contrast x Group**	F(3,15) = 0.419, p = 0.742
**Session x Contrast**	F(12,60) = 4.031, **p = 0.000**
**%CR & %FA**	
**Session**	F(4,20) = 9.719, **p = 0.000**
**Contrast (0.50, 0.75, 1.00, 1.25 octaves)**	F(3,15) = 14.535, **p = 0.000**
**Group**	F(1,5) = 0.020, p = 0.894
**Session x Group**	F(4,20) = 0.994, p= 0.433
**Contrast x Group**	F(3,15) = 1.094, p = 0.382
**Session x Contrast**	F(12,60) = 1.006, p = 0.455
**d'**	
**Session**	F(4,20) = 0.619, p = 0.654
**Contrast (0.50, 0.75, 1.00, 1.25 octaves)**	F(3,15) = 7.479, **p = 0.003**
**Group**	F(1,5) = 0.311, p = 0.601
**Session x Group**	F(4,20) = 1.737, p= 0.181
**Contrast x Group**	F(3,15) = 1.719, p = 0.206
**Session x Contrast**	F(12,60) = 3.196, **p = 0.001**
**HIT Latency**	
**Session**	F(4,20) = 2.591, p = 0.068
**Contrast (0.50, 0.75, 1.00, 1.25 octaves)**	F(3,15) = 5.232, **p = 0.011**
**Group**	F(1,5) = 0.812, p = 0.409
**Session x Group**	F(4,20) = 0.969, p= 0.446
**Contrast x Group**	F(3,15) = 0.615 p = 0.616
**Session x Contrast**	F(12,60) = 1.654, p = 0.101
**FA Latency**	
**Session**	F(4,20) = 0.251, p = 0.905
**Contrast (0.50, 0.75, 1.00, 1.25 octaves)**	F(3,15) = 2.442, p = 0.104
**Group**	F(1,5) = 6.386, p = 0.053
**Session x Group**	F(4,20) = 0.320, p= 0.861
**Contrast x Group**	F(3,15) = 0.195, p = 0.898
**Session x Contrast**	F(12,60) = 1.364, p = 0.209
**d’ comparisons (Holm-Sidak method)**	

**Group**	*p*	*p*(C 0.50 oct)	*p*(C 0.75 oct)	*p*(C 1.00 oct)	*p*(C 1.25 oct)
4.8-6.7 kHz x 8.0-11.3 kHz	0.601	0.284	0.467	0.868	0.718
**Contrast**	*p*				
0.50 x 0.75 oct	**0.040**				
0.50 x 1.00 oct	0.136				
0.50 x 1.25 oct	**0.045**				
0.75 x 1.00 oct	0.512				
0.75 x 1.25 oct	0.480				
1.00 x 1.25 oct	0.999				
**%HIT comparisons (Holm-Sidak method)**					
**Group**	*p*	*p*(C 0.50 oct)	*p*(C 0.75 oct)	*p*(C 1.00 oct)	*p*(C 1.25 oct)
4.8-6.7 kHz x 8.0-11.3 kHz	0.504	0.450	0.534	0.522	0.903
**Contrast**	*p*				
0.50 x 0.75 oct	0.934				
0.50 x 1.00 oct	0.968				
0.50 x 1.25 oct	0.979				
0.75 x 1.00 oct	0.736				
0.75 x 1.25 oct	0.888				
1.00 x 1.25 oct	1.000				
**%CR comparisons (Holm-Sidak method)**					
**Group**	*p*	*p*(C 0.50 oct)	*p*(C 0.75 oct)	*p*(C 1.00 oct)	*p*(C 1.25 oct)
4.8-6.7 kHz x 8.0-11.3 kHz	0.894	0.479	0.704	0.100	0.760
**Contrast**	*p*				
0.50 x 0.75 oct	0.671				
0.50 x 1.00 oct	**0.020**				
0.50 x 1.25 oct	**0.028**				
0.75 x 1.00 oct	0.076				
0.75 x 1.25 oct	0.082				
1.00 x 1.25 oct	1.000				

Statistical details of the frequency contrast impact on oddball responses with a STD/DED probability of 90/10% (cf., Fig. 4). 3-ways ANOVA test for repeated measures (one test per category). Statistically significant comparisons ($p < 0.05$) highlighted in bold.

Supplementary Table 6					
**%HIT & %MISS**					
**Session**	F(4,24) = 2.304, p = 0.088				
**Contrast (0.50, 0.75, 1.00, 1.25 octaves)**	F(3,18) = 6.690, **p = 0.003**				
**Group**	F(1,6) = 15.322, **p = 0.008**				
**Session x Group**	F(4,24) = 0.712, p = 0.592				
**Contrast x Group**	F(3,18) = 1.611, p = 0.222				
**Session x Contrast**	F(12,72) = 2.764, **p = 0.004**				
**%CR & %FA**					
**Session**	F(4,24) = 2.255, p = 0.093				
**Contrast (0.50, 0.75, 1.00, 1.25 octaves)**	F(3,18) = 2.891, p = 0.064				
**Group**	F(1,6) = 1.153, p = 0.324				
**Session x Group**	F(4,24) = 1.379, p = 0.271				
**Contrast x Group**	F(3,18) = 1.108, p = 0.372				
**Session x Contrast**	F(12,72) = 1.100, p = 0.374				
**d'**					
**Session**	F(4,24) = 3.468, **p = 0.023**				
**Contrast (0.50, 0.75, 1.00, 1.25 octaves)**	F(3,18) = 8.783, **p = 0.001**				
**Group**	F(1,6) = 0.121, p = 0.740				
**Session x Group**	F(4,24) = 1.168, p = 0.350				
**Contrast x Group**	F(3,18) = 1.381, p = 0.281				
**Session x Contrast**	F(12,72) = 1.535, p = 0.132				
**HIT Latency**					
**Session**	F(4,24) = 2.401, p = 0.078				
**Contrast (0.50, 0.75, 1.00, 1.25 octaves)**	F(3,18) = 0.457, p = 0.715				
**Group**	F(1,6) = 0.117, p = 0.744				
**Session x Group**	F(4,24) = 0.892, p = 0.484				
**Contrast x Group**	F(3,18) = 0.903, p = 0.459				
**Session x Contrast**	F(12,72) = 1.083, p = 0.387				
**FA Latency**					
**Session**	F(4,24) = 1.473, p = 0.242				
**Contrast (0.50, 0.75, 1.00, 1.25 octaves)**	F(3,18) = 4.572, **p = 0.015**				
**Group**	F(1,6) = 0.036, p = 0.857				
**Session x Group**	F(4,24) = 3.744, **p = 0.017**				
**Contrast x Group**	F(3,18) = 1.073, p = 0.385				
**Session x Contrast**	F(12,72) = 0.396, p = 0.961				
**d' comparisons (Holm-Sidak method)**					
**Group**	*p*	*p*(C 0.50 oct)	*p*(C 0.75 oct)	*p*(C 1.00 oct)	*p*(C 1.25 oct)

4.8-6.7 kHz x 8.0-11.3 kHz	0.740	0.993	0.422	0.406	0.769
**Contrast**	*p*				
0.50 x 0.75 oct	**0.023**				
0.50 x 1.00 oct	**0.006**				
0.50 x 1.25 oct	0.083				
0.75 x 1.00 oct	0.717				
0.75 x 1.00 oct	0.966				
1.00 x 1.25 oct	0.972				
**%HIT comparisons (Holm-Sidak method)**					
**Group**	*p*	*p*(C 0.50 oct)	*p*(C 0.75 oct)	*p*(C 1.00 oct)	*p*(C 1.25 oct)
4.8-6.7 kHz x 8.0-11.3 kHz	**0.008**	0.370	**0.010**	0.104	0.137
**Contrast**	*p*				
0.50 x 0.75 oct	1.000				
0.50 x 1.00 oct	0.250				
0.50 x 1.25 oct	**0.007**				
0.75 x 1.00 oct	0.393				
0.75 x 1.25 oct	**0.027**				
1.00 x 1.25 oct	0.997				
**%CR comparisons (Holm-Sidak method)**					
**Group**	*p*	*p*(C 0.50 oct)	*p*(C 0.75 oct)	*p*(C 1.00 oct)	*p*(C 1.25 oct)
4.8-6.7 kHz x 8.0-11.3 kHz	0.324	0.711	0.148	0.616	0.196
**Contrast**	*p*				
0.50 x 0.75 oct	**0.005**				
0.50 x 1.00 oct	0.971				
0.50 x 1.25 oct	1.000				
0.75 x 1.00 oct	0.832				
0.75 x 1.25 oct	0.148				
1.00 x 1.25 oct	0.822				

Statistical details of the frequency contrast impact on oddball responses with a STD/DEV probability of 70/30% (cf., Fig. 4). 3-ways ANOVA test for repeated measures (one test per category). Statistically significant comparisons ($p < 0.05$) highlighted in bold.

**Supplementary Table 7**	
**d' Contrast 0.50 octaves**	
**Session**	F(4,20) = 3.731, **p = 0.020**
**STD/DEV Probability (90/10, 70/30 %)**	F(1,5) = 34.272, **p = 0.002**
**Group**	F(1,5) = 1.478, p = 0.278
**Session x Group**	F(4,20) = 2.071, p = 0.123
**STD/DEV Probability x Group**	F(1,5) = 1.219, p = 0.320
**Session x STD/DEV Probability**	F(4,20) = 1.102, p = 0.383
**d' Contrast 0.75 octaves**	
**Session**	F(4,20) = 0.492, p = 0.742
**STD/DEV Probability (90/10, 70/30 %)**	F(1,5) = 17.124, **p = 0.009**
**Group**	F(1,5) = 1,616, p = 0.260
**Session x Group**	F(4,20) = 0.327, p = 0.857
**STD/DEV Probability x Group**	F(1,5) = 0.075, p = 0.795
**Session x STD/DEV Probability**	F(4,20) = 1.854, p = 0.158
**d' Contrast 1.00 octaves**	
**Session**	F(4,20) = 0.911, p = 0.477
**STD/DEV Probability (90/10, 70/30 %)**	F(1,5) = 29.138, **p = 0.003**
**Group**	F(1,5) = 0.096, p = 0.770
**Session x Group**	F(4,20) = 0.399, p = 0.807
**STD/DEV Probability x Group**	F(1,5) = 0.560, p = 0.488
**Session x STD/DEV Probability**	F(4,20) = 0.970, p = 0.446
**d' Contrast 1.25 octaves**	
**Session**	F(4,20) = 5.383, **p = 0.004**
**STD/DEV Probability (90/10, 70/30 %)**	F(1,5) = 47.680, **p = 0.001**
**Group**	F(1,5) = 0.336, p = 0.587
**Session x Group**	F(4,20) = 2.504, p = 0.075
**STD/DEV Probability x Group**	F(1,5) = 0.007, p = 0.935
**Session x STD/DEV Probability**	F(4,20) = 3.392, **p = 0.028**

Statistical details of the probability comparison for the different frequency contrast tested (cf., Fig. 4). 3-ways ANOVA-test for repeated measures (one test per category). Statistically significant comparisons ($p < 0.05$) highlighted in bold.

**Supplementary Table 8**	
	**%HIT & %MISS**
**Session**	F(4,20) = 0.394, p = 0.746
**Group**	F(1,5) = 1.652, p = 0.255
**Session x Group**	F(4,20) = 0.407, p = 0.737
	**%CR & %FA**
**Session**	F(4,20) = 0.558, p = 0.696
**Group**	F(1,5) = 5.113, p = 0.073
**Session x Group**	F(4,20) = 0.569, p = 0.688
	**d'**
**Session**	F(4,20) = 0.689, p = 0.608
**Group**	F(1,5) = 0.253, p = 0.637
**Session x Group**	F(4,20) = 0.411, p = 0.799
**d' comparissons by sequence**	
	p
Many-deviant x Oddball Paradigm C0.50 oct.	0.999
Many-deviant x Oddball Paradigm C0.75 oct.	1.000
Many-deviant x Oddball Paradigm C1.00 oct.	0.270
Many-deviant x Oddball Paradigm C1.25 oct.	**0.030**

Statistical details of the many-deviant task responses with a STD/DEV Probability of 90/10% (cf., Fig. 5). 2-way ANOVA test (one test per category). Statistically significant comparisons ($p < 0.05$) highlighted in bold.

Supplementary Table 9	
	**%HIT & %MISS**
**Session**	F(4,24) = 1.512, p = 0.230
**Group**	F(1,6) = 1.468, p = 0.271
**Session x Group**	F(4,24) = 0.784, p = 0.547
	**%CR & %FA**
**Session**	F(4,24) = 0.563, p = 0.691
**Group**	F(1,6) = 6.860, **p = 0.040**
**Session x Group**	F(4,24) = 0.702, p = 0.105
	**d'**
**Session**	F(4,24) = 2.073, p = 0.116
**Group**	F(1,6) = 1.062, p = 0.343
**Session x Group**	F(4,24) = 0.414, p = 0.797

Statistical details of the many-deviant task responses with a STD/DEV Probability of 70/30% (cf., Fig. 5). 2-way ANOVA test (one test per category). Statistically significant comparisons ($p < 0.05$) highlighted in bold.

Supplementary Table 10						
	**Group 1: 4.8-6.7 kHz**			**Group 2: 8.0-11.3 kHz**		
	Task	Contrast	ISI	Task	Contrast	ISI
1º	Oddball sequence	0.50 oct	1.5 s	Oddball sequence	0.50 oct	1.5 s
2º	Deviant frequency contrast variation	0.75 oct	1.5 s	Oddball sequence	0.50 oct	2 s
3º	Oddball sequence	0.50 oct	2 s	Deviant frequency contrast variation	0.75 oct	1.5 s
4º	Deviant frequency contrast variation	1.00 oct	1.5 s	Oddball sequence	0.50 oct	4 s
5º	Oddball sequence	0.50 oct	4 s	Deviant frequency contrast variation	1.00 oct	1.5 s
6º	Many-deviant	N/A	1.5 s	Many-deviant	N/A	1.5 s
7º	Deviant frequency contrast variation	1.25 oct	1.5 s	Deviant frequency contrast variation	1.25 oct	1.5 s

Order of presentation of the different behavioural tasks for the STD/DEV probability of 90/10 %.
