# Supplementary material for: Novelty detection in an auditory oddball task on freely moving rats
Source: Commun Biol. 2023 Oct 19;6:1063. doi: 10.1038/s42003-023-05403-y (PMC10587131; doi:10.1038/s42003-023-05403-y)
Supplement: Supplementary file 2 — Description of Additional Supplementary Files [file 42003_2023_5403_MOESM2_ESM.pdf]

Description of Additional Supplementary Files

File name: Supplementary Data 1
Description: Data set for figures.
