# Supplementary material for: Novelty detection in an auditory oddball task on freely moving rats
Source: Commun Biol. 2023 Oct 19;6:1063. doi: 10.1038/s42003-023-05403-y (PMC10587131; doi:10.1038/s42003-023-05403-y)
Supplement: Supplementary file 4 — Reporting Summary [file 42003_2023_5403_MOESM4_ESM.pdf]

nature portfolio

Corresponding author(s): Manuel S. Malmierca

Last updated by author(s): Sep 27, 2023

Reporting Summary

Nature Portfolio wishes to improve the reproducibility of the work that we publish. This form provides structure for consistency and transparency in reporting. For further information on Nature Portfolio policies, see our Editorial Policies and the Editorial Policy Checklist.

Statistics

For all statistical analyses, confirm that the following items are present in the figure legend, table legend, main text, or Methods section.

n/a	Confirmed	
☐	☒	The exact sample size (*n*) for each experimental group/condition, given as a discrete number and unit of measurement
☐	☒	A statement on whether measurements were taken from distinct samples or whether the same sample was measured repeatedly
☐	☒	The statistical test(s) used AND whether they are one- or two-sided<br>*Only common tests should be described solely by name; describe more complex techniques in the Methods section.*
☐	☒	A description of all covariates tested
☐	☒	A description of any assumptions or corrections, such as tests of normality and adjustment for multiple comparisons
☐	☒	A full description of the statistical parameters including central tendency (e.g. means) or other basic estimates (e.g. regression coefficient) AND variation (e.g. standard deviation) or associated estimates of uncertainty (e.g. confidence intervals)
☐	☒	For null hypothesis testing, the test statistic (e.g. *F*, *t*, *r*) with confidence intervals, effect sizes, degrees of freedom and *P* value noted<br>*Give P values as exact values whenever suitable.*
☒	☐	For Bayesian analysis, information on the choice of priors and Markov chain Monte Carlo settings
☒	☐	For hierarchical and complex designs, identification of the appropriate level for tests and full reporting of outcomes
☒	☐	Estimates of effect sizes (e.g. Cohen's *d*, Pearson's *r*), indicating how they were calculated

Our web collection on statistics for biologists contains articles on many of the points above.

Software and code

Policy information about availability of computer code

Data collection	ABR responses were processed with BioSig software (Tucker-Davis Technologies).<br>All behavioural experiments were carried out in a Med Associates operational cage, controlled by custom-made scripts (written in Trans V software by Cibertec, S.A., Madrid, Spain). Responses were automatically quantified by software MED-PC V version 5.1 (Med Associates, Inc.).
Data analysis	All figures were obtained using MATLAB R2022A.<br>The statistical analysis for ABR recordings and the data derived from behaviour tasks was performed using SPSS v.28 (IBM, Armonk, NY, United States).

For manuscripts utilizing custom algorithms or software that are central to the research but not yet described in published literature, software must be made available to editors and reviewers. We strongly encourage code deposition in a community repository (e.g. GitHub). See the Nature Portfolio guidelines for submitting code & software for further information.

Data

Policy information about availability of data

All manuscripts must include a data availability statement. This statement should provide the following information, where applicable:

- Accession codes, unique identifiers, or web links for publicly available datasets
- A description of any restrictions on data availability
- For clinical datasets or third party data, please ensure that the statement adheres to our policy

The datasets generated during the current study are available from the corresponding author on reasonable request and at GREDOS repository from the Salamanca University with the following identifier: http://hdl.handle.net/10366/153079. The source data for the graphs and charts in the manuscript can be found in Supplementary Data 1.

Research involving human participants, their data, or biological material

Policy information about studies with human participants or human data. See also policy information about sex, gender (identity/presentation), and sexual orientation and race, ethnicity and racism.

Reporting on sex and gender	This affirmation has not been collected.
Reporting on race, ethnicity, or other socially relevant groupings	This affirmation has not been collected.
Population characteristics	This affirmation has not been collected.
Recruitment	This affirmation has not been collected.
Ethics oversight	This affirmation has not been collected.

Note that full information on the approval of the study protocol must also be provided in the manuscript.

Field-specific reporting

Please select the one below that is the best fit for your research. If you are not sure, read the appropriate sections before making your selection.

☒ Life sciences ☐ Behavioural & social sciences ☐ Ecological, evolutionary & environmental sciences

For a reference copy of the document with all sections, see nature.com/documents/nr-reporting-summary-flat.pdf

Life sciences study design

All studies must disclose on these points even when the disclosure is negative.

Sample size	Sample size was determined using Epidat 4.2 (a free statistical platform developed by Hervada Vidal et al., Rev Esp Salud Pública, 2004; 78(2):277-80). We performed an independent samples test: equality of variances, standardized mean difference of 1.75, statistical confidence level of 95.0% and statistical power of 80%.
Data exclusions	No data were excluded from the analysis.
Replication	To verify the reproducibility of the experimental findings, we assigned 4 animals to each experimental group from different litters and data were collected along 5 consecutive seassions for each paradigm
Randomization	Subjects were assigned randomly to each experimental group.
Blinding	Both the application of the behavioral protocol and data collection was automatic, without researcher interaction.

Reporting for specific materials, systems and methods

We require information from authors about some types of materials, experimental systems and methods used in many studies. Here, indicate whether each material, system or method listed is relevant to your study. If you are not sure if a list item applies to your research, read the appropriate section before selecting a response.

Materials & experimental systems

n/a	Involved in the study
☒	☐ Antibodies
☒	☐ Eukaryotic cell lines
☒	☐ Palaeontology and archaeology
☐	☒ Animals and other organisms
☒	☐ Clinical data
☒	☐ Dual use research of concern
☒	☐ Plants

Methods

n/a	Involved in the study
☒	☐ ChIP-seq
☒	☐ Flow cytometry
☒	☐ MRI-based neuroimaging

Animals and other research organisms

Policy information about studies involving animals; ARRIVE guidelines recommended for reporting animal research, and Sex and Gender in Research

Laboratory animals	Female Long Evans rats aged 7-10 weeks old
Wild animals	The study did not involve wild animals.
Reporting on sex	The experiments were carried out only on females to maintain uniformity of results, given the small number of subjects.
Field-collected samples	The study did not involve samples collected from the field.
Ethics oversight	All experimental procedures were carried out at the University of Salamanca and all methodological procedures were approved by the Bioethics Committee for Animal Care of the University of Salamanca (USAL-ID-574 and 683) and performed in compliance with the standards of the European Convention ETS 123, the European Union Directive 2010/63/EU, and the Spanish Royal Decree 53/2013 for the use of animals in scientific research.

Note that full information on the approval of the study protocol must also be provided in the manuscript.
